# An efficient multifunction fMRI localizer for high-level visual, auditory, and cognitive regions in humans

**DOI:** 10.1162/IMAG.a.905

**Published:** 2025-10-14

**Authors:** Ammar I. Marvi, Sam Hutchinson, Evelina Fedorenko, Rebecca R. Saxe, Frederik S. Kamps, Tamar I. Regev, Emily M. Chen, Nancy G. Kanwisher

**Affiliations:** Department of Psychology, Harvard University, Cambridge, MA, United States; Department of Brain & Cognitive Sciences, Massachusetts Institute of Technology, Cambridge, MA, United States; McGovern Institute for Brain Research, Massachusetts Institute of Technology, Cambridge, MA, United States; Department of Psychology, University of Edinburgh, Edinburgh, United Kingdom; Department of Psychology, Stanford University, Stanford, CA, United States

**Keywords:** human brain, functional regions of interest and functional localization

## Abstract

One of the most robust findings in human cognitive neuroscience is the discovery that many regions of the cortex are engaged in distinctive, often very specific, functions. Although these regions are found in approximately the same location in almost all typical participants, their exact location varies from one individual to the next. Thus, the first step in studying these regions is to identify them in each participant individually. Standard functional localizers have been devised to accomplish this goal, but most localizers identify only a few regions. Many important questions in modern neuroscience can only be answered by measuring the responses of multiple cortical regions at the same time. Here, we introduce a new Efficient Multifunction fMRI localizer (EMFL) in which visual and auditory conditions are presented simultaneously, enabling the identification, in just 14 minutes of fMRI scan time, of 14 of the most widely-studied cortical regions: those selectively engaged in perceiving faces, places, bodies, words, objects, and speech sounds; understanding language and other people’s thoughts; and engaging broadly in demanding cognitive tasks (the “multiple demand” system). We validate the EMFL by showing that it identifies the major functional regions of interest as well as the standard localizers do, in a quarter of the scan time. The stimuli and presentation code for this new localizer are publicly available, enabling future studies to efficiently identify the major functional regions of interest with the same procedure across labs.

## 1 Introduction

The last few decades of research in human cognitive neuroscience have revealed the functional organization of the human brain in considerable detail. Prominent in this organization is a set of cortical regions with functionally distinctive and often highly specific response profiles that are present in approximately the same location in virtually every neurotypical participant. The most robust and widely replicated of these regions respond selectively to visual images of faces, places, bodies, or text, or to auditory clips containing speech sounds, or during high-level cognitive operations like understanding language or thinking about other people’s thoughts. Other regions of the parietal and frontal lobes are distinguished by their lack of functional specificity, that is, by their broad engagement across multiple cognitively demanding tasks. The existence of this highly systematic functional organization of the cortex invites a deeper set of questions into the representations, computations, connectivity, cytoarchitecture, development, and evolution of these regions, as well as the interactions among them and their possible alterations in clinical disorders. To approach these questions, the first step is to find these regions in new participants. There is just one problem.

Although each of these functionally distinctive cortical regions is found in approximately the same location across individuals, their exact location varies considerably from brain to brain. As a result, purely anatomical landmarks are insufficient to precisely identify the location of these regions in new participants. The only way to identify the exact location and extent of any of these regions in a particular individual is to scan them with fMRI on a “localizer” scan that includes the functional contrast by which that region or set of regions is defined (e.g., a higher response to images of faces than objects in the lateral fusiform gyrus identifies the fusiform face area). For this purpose, standard localizer contrasts and paradigms have been developed to identify these regions, enabling a cumulative research program in which studies across participants and labs can refer to the same region because it is identified by the same functional contrast. However, many scientific questions can only be answered by measuring responses of many of these regions at once. Running the standard localizer scans to identify a wide selection of the most-commonly studied regions can take upwards of an hour, placing a significant burden on both the participant’s patience and the PI’s budget. Here, we present a new Efficient Multifunction Localizer (EMFL) that enables robust localization of all of the regions with the functional selectivities listed above in each individual participant in as little as 14 minutes of scan time.

**Fig. 1.**
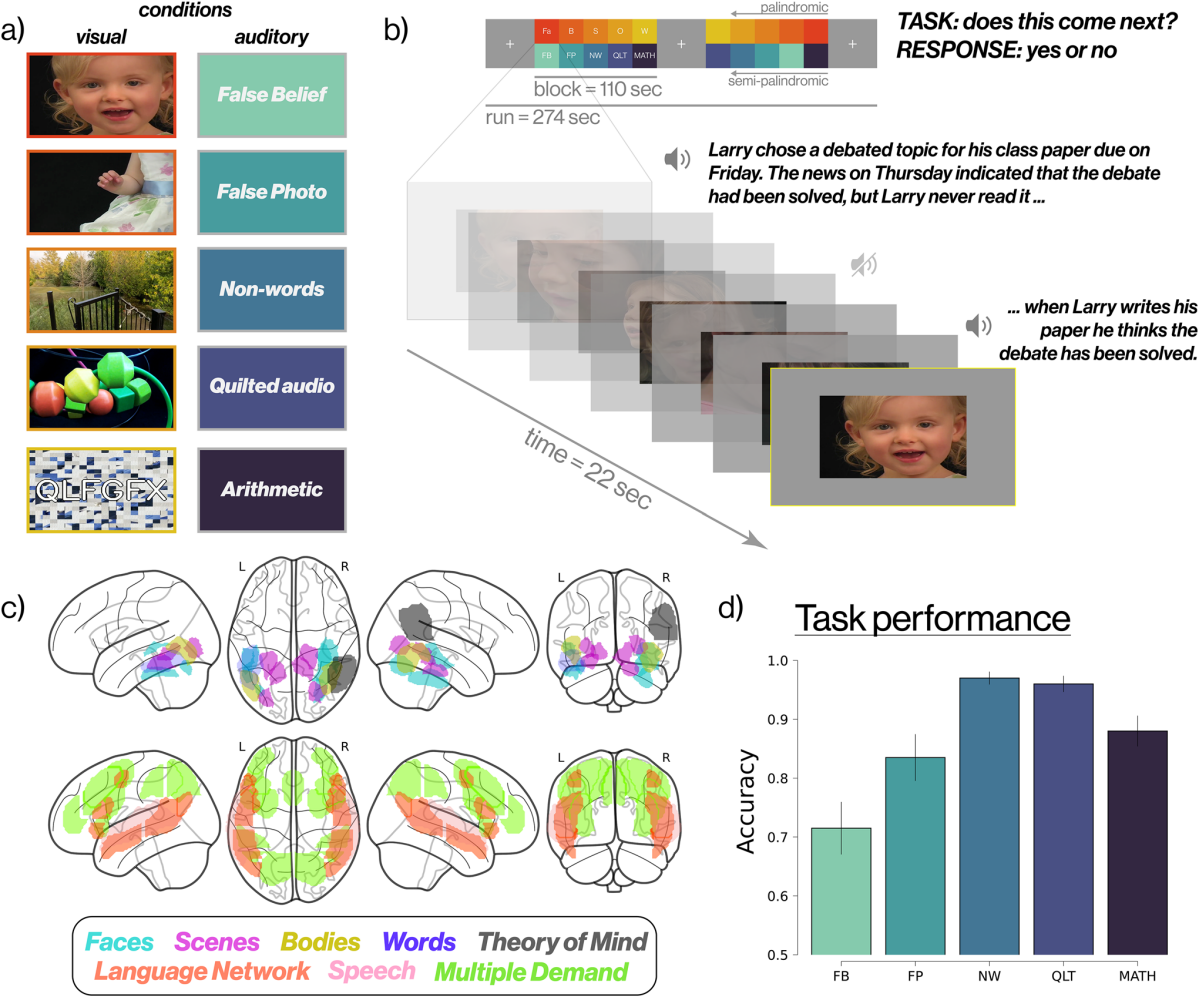
Design of the Efficient Multifunction Localizer (EMFL). (a) the five visual (left) and five auditory (right) conditions used in EMFL. (b) blocked structure of an example run (top) and presentation of stimuli within a block (bottom). Each block implements one visual and one auditory condition; participants perform a ‘*Does this come next?’* task only on the auditory stimuli. Across five runs of the experiment, each combination of visual and auditory conditions occurs twice, unconfounding the visual and auditory conditions. (c) brain regions targeted by the EMFL; “glass brains” show the anatomical constraint parcels used for each contrast. (d) accuracy on the ‘Does this come next?’ task, by auditory condition. *Condition abbreviations: Faces (Fa), Scenes (S), Bodies (B), Objects (O), Words/scrambled (W), False belief (FB), False photo (FP), Non-words (NW), Quilted audio (QLT), Arithmetic (MATH).*

To accomplish this, we implement a blocked design (Fig. 1) in which participants watch sets of short video clips from five different visual categories (faces, bodies, scenes, objects, and words), while simultaneously listening to and performing unrelated tasks on five different kinds of audio stimuli (false belief stories, false photo stories, arithmetic problems, non-word strings, and ‘quilted’ or scrambled, speech). We counterbalance the pairing of each visual-auditory combination across five runs to unconfound the responses to stimuli in one modality from responses to the other. We then assess the efficacy of this new localizer by testing whether it reliably identifies voxels whose selectivity cross-validates with held-out data from the EMFL and with the standard localizers for these regions. We find that the EMFL identifies similar fROIs with similar response profiles to those observed in standard localizers, thereby enabling the main functional regions to be identified in a unified and short localizer scan.

## 2 Methods

This study was approved by and in full compliance with the WCG IRB Connexus board. The methods and analyses of this study were pre-registered on OSF (materials available here: https://doi.org/10.17605/OSF.IO/GJSDB) prior to data collection and analysis. Data from an initial pilot phase (15 subjects, 9 females) were collected and analyzed to refine the experimental design prior to pre-registration. Scripts for the EMFL and relevant analyses are available at these GitHub repositories: https://github.com/aimarvi/efficient_localizer/ (localizer) and https://github.com/aimarvi/emfl_analysis (analysis). fMRI data are available on OpenNeuro: https://openneuro.org/datasets/ds006179/versions/1.0.1.

### 2.1 EMFL design

The EMFL uses a blocked design in which five visual conditions are crossed with five unrelated but simultaneously-presented auditory conditions (see Fig. 1). Tasks are performed only on the auditory stimuli, on the hypothesis (validated here) that visual category-selective regions show selective activation for their preferred stimuli even when participants are conducting a demanding orthogonal task on auditory stimuli. Visual conditions consist of dynamic videos of 1) faces, 2) scenes, 3) objects, 4) body parts (excluding faces), and 5) six-letter consonant strings superimposed on a scrambled-object background. Auditory conditions (Table 1) consist of 1) stories describing false beliefs, 2) stories describing a false physical representation (“false photo” stories), 3) spoken non-word strings, 4) synthesized audio quilts (Overath et al., 2015) of the non-word strings, and 5) arithmetic problems.

**Table 1.** Example stimuli of the EMFL auditory task.

Condition	Task	First statement	Second statement
False belief stories	Is the second statement consistent with the first?	Laura didn’t have time to braid her horse’s mane before going to camp. While she was at camp, William braided the horse’s mane for her.	Laura returns assuming that her horse’s mane is braided.
False photo stories	Is the second statement consistent with the first?	The traffic camera snapped an image of the black car as it sped through the stoplight. Soon after, the car was painted red.	According to the traffic camera, the car is black.
Non-word strings	Is the gender of the second speaker the same as the first speaker?	(in female voice) Alf nimes beas brole inca coaker nour tunt ang drare sha doga. bave heers titer, sha bagners wole uld ake weps rho freir coaker fom breekmost.	(in female voice) Sha bawners ank surskills tu fand sha somo sussing fror sha coaker.
Audio quilts	Is the gender of the second speaker the same as the first speaker?	(in female voice) *unintelligible audio*	(in male voice) *unintelligible audio*
Arithmetic	Is the given solution to the equation correct?	Nine. times three. plus three. minus six. divided by eight. times two	Equals six.

Auditory stimuli occurred in five Conditions. Participants performed variants of a *‘Does this come next?’* Task, comprising a First statement followed by a Second statement. Correct responses (top to bottom): no, yes, yes, no, yes.

Each of the 25 possible combinations of a visual and an auditory condition occurs twice across five runs, with each run lasting 274 seconds (ten 22-second stimulus blocks and three 18-second fixation blocks). Each condition occurs twice per run in palindromic (for visual conditions) or semi-palindromic (for auditory conditions) order. A single block consists of a visual component and an auditory component presented simultaneously. The visual component consists of seven 3-second clips of a single visual condition (e.g., videos of human faces). Face, body, and object videos were taken from a widely used dynamic localizer (Pitcher et al., 2011); scene videos were egocentric navigation videos selected as those producing the strongest response in the three scene-selective regions in a prior study (Chen et al., 2024); and words were superimposed on grid-scrambled versions of the object videos. At the same time, a single exemplar from an auditory condition is played for 20.5 seconds, consisting of a long main clip and a second, shorter clip, followed by a silent response period of 1.5 seconds.

Participants are instructed to fixate on the visual stimuli while performing a *‘Does this come next?’* task on the auditory stimuli. After listening to the main clip of audio, subjects hear a short continuation of the clip and are asked to decide whether the second clip is consistent with the first, responding yes/no via a button box. The exact meaning of ‘Does this come next?’ varies depending on the content of the auditory stimulus. Example stimuli are shown in Table 1. A yellow outline appears around the edges of the screen indicating the time in which participants can submit their response to the ‘Does this come next?’ task and remains present for the duration of the response period.

### 2.2 Experimental data collection

We scanned 20 participants, each over two imaging sessions, on the EMFL as well as a battery of standard functional localizers and anatomical scans. The details of the standard localizers—including targeted fROIs, task, and stimuli—are described in Table 2. The standard visual category localizer we used contains static images of faces, objects, scenes, and scrambled objects, aka “FOSS” (Epstein & Kanwisher, 1998), but not bodies or words. We, thus, created our own additional localizer for the EBA and VWFA that contained blocks of words, line drawings of bodies, and line drawings of objects. Participants were familiarized with the stimuli and task in our EMFL localizer by performing a short practice run outside the scanner before their scanning session.

**Table 2.** Overview of standard functional localizers used here.

Standard localizer	Targeted fROI(s)	Stimuli	Task	Duration
Epstein and Kanwisher (1998)	FFA, OFA, fSTS, PPA, OPA, RSC, LOC	Static images of faces, scenes, objects, and scrambled objects	Button press after one-back presentation	16 minutes
Fedorenko et al. (2010)	High-level language processing regions	Written English sentences and nonword sequences	Button press after on-screen indication	10 minutes
Jacoby et al. (2016)	rTPJ	Written stories of characters experiencing physical or emotional pain	Button press (1–4) rating main character’s pain or suffering	10 minutes
Fedorenko et al. (2013)	Multiple demand network (frontal and parietal MD)	Sequences of visual spatial working memory grids	Button press choosing correct grid pattern	14 minutes
Regev et al., in prep.	Speech processing regions	Spoken non-words and scrambled audio	Button press after on-screen indication	4 minutes
Newly developed for this study	EBA, VWFA	Static black & white line drawings of bodies, objects, and words	Button press after one-back presentation	18 minutes

Battery of standard functional localizers that have been used across numerous prior studies, as well as two newer localizers used here to validate the EMFL. *fROI abbreviations: fusiform face area (FFA), occipital face area (OFA), faceselective regions of the superior temporal sulcus (fSTS), parahippocampal place area (PPA), occipital place area (OPA), retrosplenial cortex (RSC), lateral occipital complex (LOC), right (rTPJ) and left (lTPJ) temporal parietal junction, right STS (rSTS), medial prefrontal cortex (mPFC), extrastriate body area (EBA), visual word form area (VWFA).*

Participants were recruited from the Massachusetts Institute of Technology and the Greater Boston area (n = 20, 10 females 18–50 years old). All participants provided written informed consent of the protocol approved by the Allen Institute for Brain Science WCB IRB Connexus and were subsequently compensated $75 for each 2-hour scan session. Data from five additional participants were excluded from analyses because they did not return for a second scanning session.

### 2.3 fMRI data acquisition and preprocessing

All imaging was performed on a Siemens 3T MAGNETOM Prisma scanner with a 32-channel head coil at the Athinoula A. Martinos Imaging Center at MIT. For each subject, a high-resolution T1-weighted anatomical image (MPRAGE: TR = 2.53 seconds; TE = 3.57 ms; α = 9°; FOV = 256 mm; matrix = 256 × 256; slice thickness = 1 mm; 176 slices; acceleration factor = 3; 24 reference lines) was collected in addition to whole-brain functional data using a T2-weighted echo planar imaging pulse sequence (TR = 2 seconds; TE = 30 ms; α = 90°; FOV = 208 mm; matrix = 104 × 104; slice thickness = 2 mm; voxel size = 2 × 2 mm in-plane; slice gap = 0 mm; 52 slices).

fMRI data were analyzed using the FreeSurfer Functional Analysis Stream (FS-FAST) software (version 6.0.0) and run via custom MATLAB (version 2018b) scripts. The native anatomical space was first reconstructed for each participant using the standard FS-FAST pipeline. Pre-processing of functional data included motion correction, smoothing with a 3 mm FWHM Gaussian kernel, binary brain mask creation, intensity normalization, and registration to the native anatomical space with 6 degrees of freedom.

**Fig. 2.**
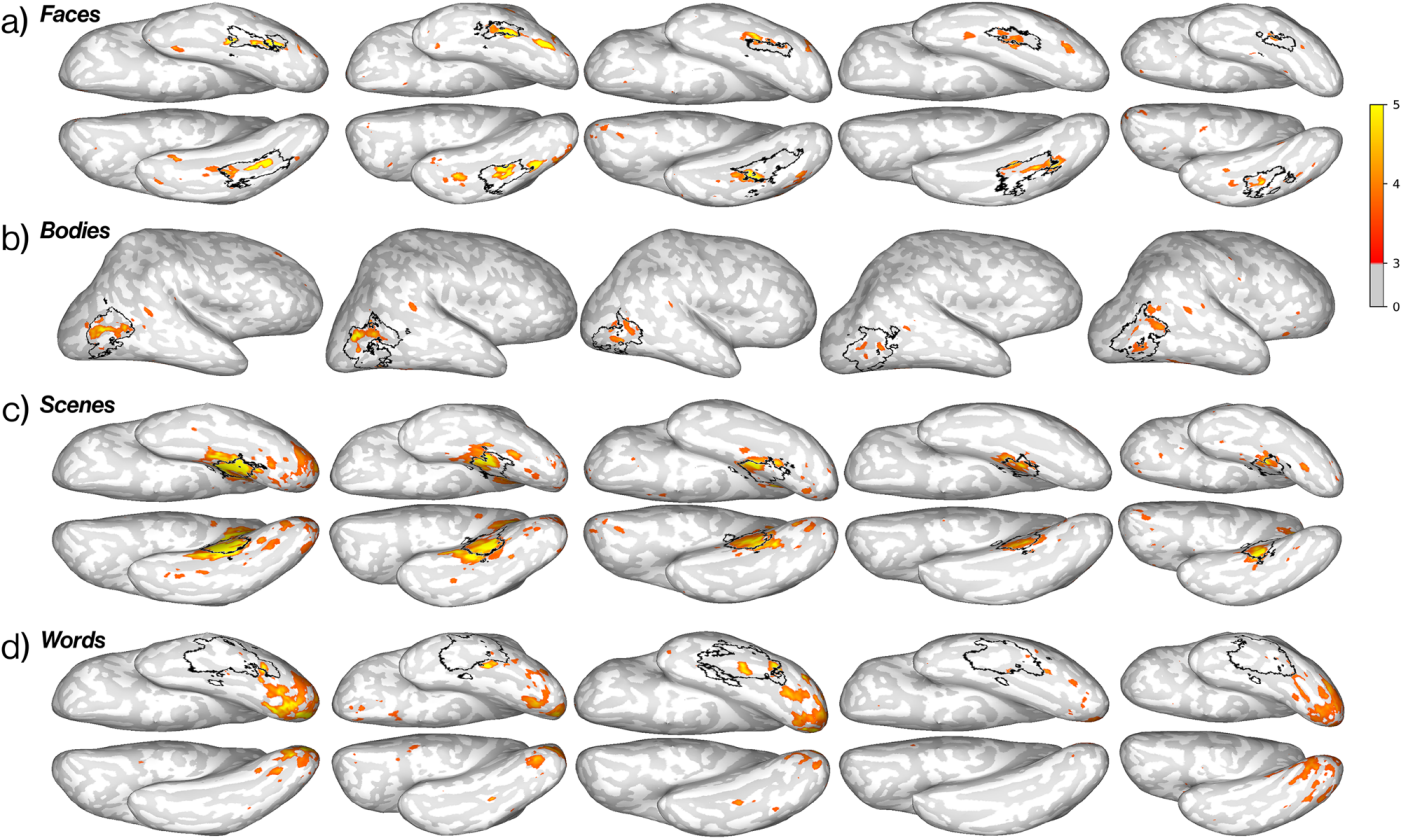
EMFL captures expected functional topography in visually selective regions. Brain activation maps of four visual contrasts in the same five participants using data from all five runs of the EMFL. Each brain shows the functional contrast for a given condition as −*log*(*p*-*value*) ∗ *sgn*(*t*-*test*), thresholded at +3. Surface projections of relevant anatomical constraint parcels are outlined in black. Contrasts: (a) Faces > Objects, (b) Scenes > Objects, (c) Bodies > Objects, and (d) Words > Objects. Although fROI-based analyses of these regions were conducted in only one hemisphere per region (see Table 3), ventral surface views here show activations in both hemispheres. Analyses were conducted in the volume and are here projected to the surface for visualization only.

**Fig. 3.**
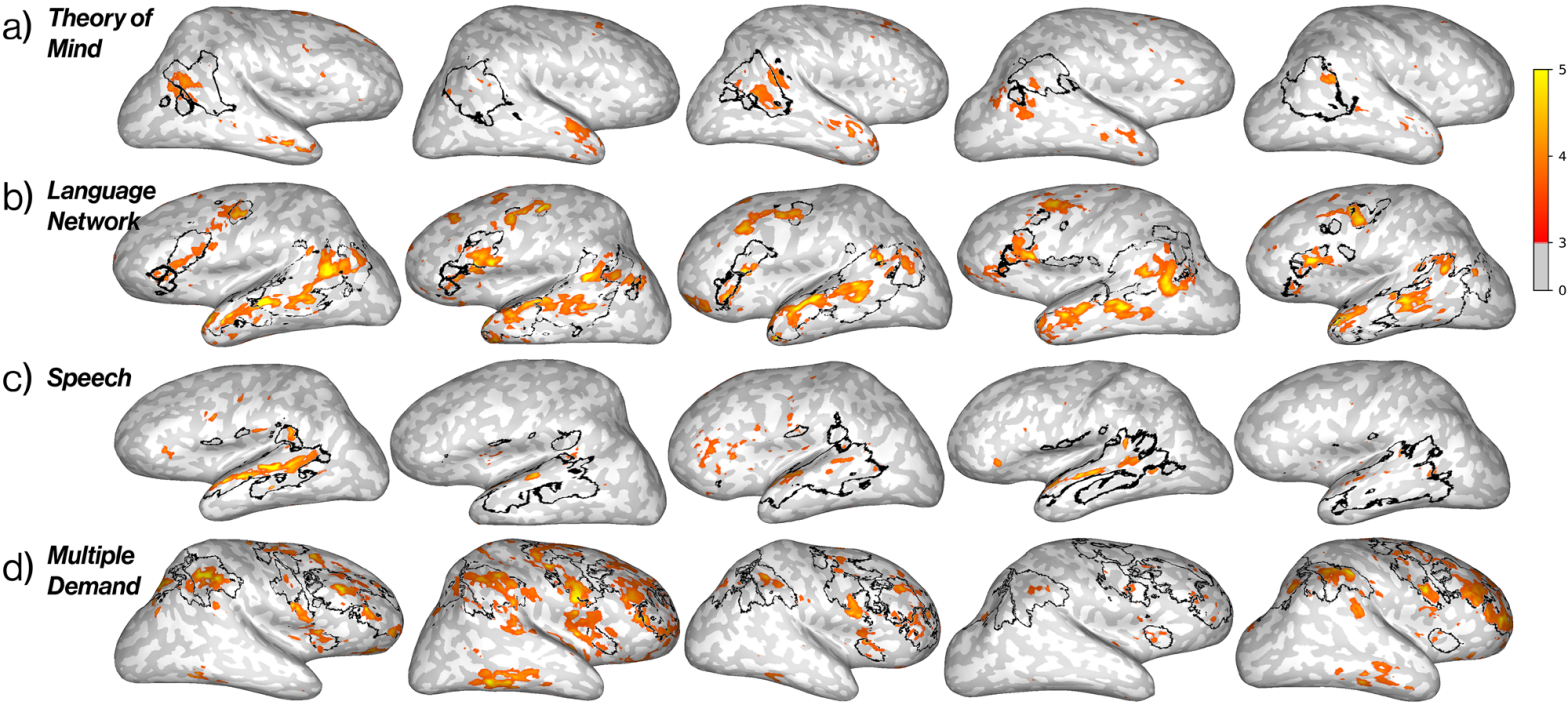
EMFL captures expected functional topography in speh and higher-level processing regions. Brain activation maps of five auditory contrasts in five representative subjects after five runs of the EMFL, plotted in the individual’s native space. Each brain shows the functional contrast for a given condition as −*log*(*p*-*value*) ∗ *sgn*(*t*-*test*), thresholded above +3. Surface projections of relevant anatomical parcels are outlined in black. Contrasts: (a) False belief > False photo, (b) English > Nonwords, (c) Non-words > Quilted audio, and (d) Arithmetic > English. Analyses were conducted in the volume and are here projected to the surface for visualization only.

First-level analysis of functional data was performed in the native space of each participant for each experiment. Conditions were included as covariates of interest along with six nuisance regressors for motion correction in a general linear model (GLM), modeled as a boxcar function convolved with a canonical hemodynamic response function (HRF). Functional contrasts—including t- and F- statistics, significance values, and effect sizes—were then estimated using the fitted GLM in the FS-FAST first-level analysis pipeline, which constructs design and contrast matrices, concatenates all functional runs, fits regression coefficients in the model to the voxel-wise time course, and computes significance values (Fischl, 2012; https://surfer.nmr.mgh.harvard.edu/fswiki/FsFast). The following contrasts were computed for the visual stimuli: Faces > Objects, Scenes > Objects, Bodies > Objects, Words/scrambled > Objects, and Objects > Words/scrambled (n.b. words were superimposed on a background of scrambled object videos to maximize stimulus utility). The following contrasts were computed for the auditory stimuli: False belief > False photo, English > Non-words, Non-words > Quilted audio, and Arithmetic > English. The ‘English’ condition was created by averaging over responses to both the ‘False belief’ and ‘False photo’ stimuli but not the ‘Arithmetic’ stimuli. See Figures 2 and 3 for whole-brain activation maps in example participants.

### 2.4 Definition of functional regions of interest (fROIs)

Functional regions of interest (fROIs) were constrained to lie within previously published anatomical parcels (see Fig. 1C for illustration and Table 3 for details). We first transformed the parcels into each participant’s native space, and then selected the top 10% of voxels with the strongest significance for the relevant contrast within each parcel. First, we defined these fROIs using all five runs of the EMFL for maximum statistical power. Next, we used the three odd-numbered runs to test whether three runs were sufficient to localize the fROIs and to use held-out data to test the selectivity of the responses.

**Table 3.** fROIs targeted in this study.

Froi	Hemi.	Source of parcel	EMFL contrast	Standard contrast	Processing domain
FFA, OFA, fSTS	Right	Julian et al. (2012)	Faces > Objects	Faces > Objects	Faces
PPA, OPA, RSC	Right	Julian et al. (2012)	Scenes > Objects	Scenes > Objects	Scenes
LOC	Right	Julian et al. (2012)	Objects > Words/scrambled	Objects > Scrambled	Non-specific objects
EBA	Right	Julian et al. (2012)	Bodies > Objects	Bodies > Objects	Bodies
VWFA	Left	Saygin et al. (2016)	Words/scrambled > Objects	Words > Objects	Visual word forms
Rtpj	Right	Dufour et al. (2013)	False belief > False photo	Emotional pain > Physical pain	Social cognition (Theory of Mind)
Language network (IFGorb, IFG, MFG, AntTemp, Post-Temp, AG)	Left	Fedorenko et al. (2010)	English (false belief + false photo) > Non-words	Sentences > non-words	Language (word re-trieval, syntax)
STG	Bilateral	Regev et al., in prep.	Non-words *>* Quilted audio	Non-words *>* Degraded speech	Speech (phonemes)
Frontal MD (IFGop, SFG,MFG1, MFG2, MFGorb)	Bilateral	Fedorenko et al. (2013)	Arithmetic *>* English (false belief + false photo)	Hard *>* Easy	Domain-general executive demands
Parietal MD (APG, PPG,MPG)	Bilateral	Fedorenko et al. (2013)	Arithmetic *>* English (false belief + false photo)	Hard *>* Easy	Domain-general executive demands

Functional regions of interest (fROIs) targeted by the EMFL (first column), source of anatomical parcels used to constrain fROI bounds (second column), functional contrast of stimulus conditions used in the EMFL to define an fROI (third column), and the comparable contrast used in standard localizers to define the same fROI (fourth). Processing domain in which the fROI is involved (fifth). *fROI abbreviations: inferior and middle frontal gyrus (IFG and MFG), orbital region of IFG (IFGorb), anterior and posterior temporal areas (AntTemp and PostTemp), angular gyrus (AG), superior temporal gyrus (STG), opercular region of IFG (IFGop), superior and middle frontal gyrus (SFG, MFG1 and MFG2), orbital region of MFG (MFGorb), anterior, middle, and posterior parietal cortex (APG, MPG, and PPG). See Tables 2 and 3 legend for additional abbreviations.*

As noted in our pre-registration, it was not clear in advance whether the Theory of Mind contrast (False belief > False photo) would be stronger when analyzing the full block, or only the second half of the block (which contained more mental state content). We, therefore, tried both and found that using the full block worked marginally better, and used that for all analyses reported here.

### 2.5 Estimating fROI selectivity

**Fig. 4.**
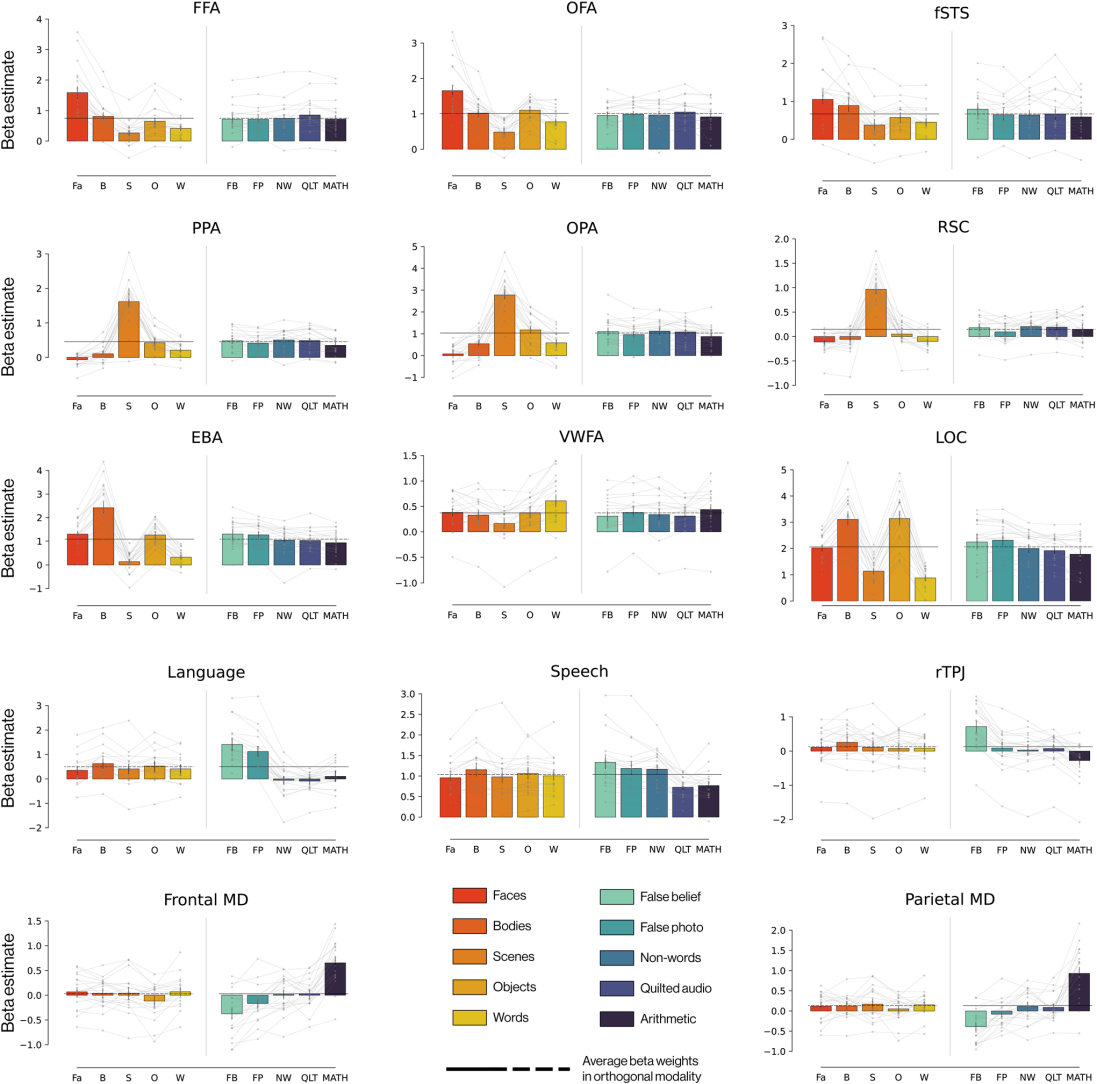
EMFL-defined fROIs are functionally specific for their preferred condition. Response of each functional region of interest defined by EMFL to each of the 10 conditions in EMFL. We selected voxels by their functional selectivity during even numbered runs (run 2, 4) and measured their responses in three held-out runs (run 1, 3, 5) and vice versa; bar charts here show the average response across the two even-odd splits of the data, averaged across the 20 participants. Horizontal lines indicate the average response across the five conditions in the modality used to define the region (solid black portion), used as the baseline (dotted black portion) against which to consider responses in the modality not used to define the region. *See Figure 1 and Tables 2 to 3 legend for condition and ROI abbreviations.*

To test whether the EMFL successfully identified fROIs with the expected selectivities, we first defined each fROI as described above using just the three odd-numbered runs. We then measured the response of this fROI to each of the 10 conditions (5 visual and 5 auditory) using the held-out data from the even-numbered runs. This procedure was repeated using the even-numbered runs to select voxels and the odd runs to measure responses in these voxels. The two estimates were then averaged to derive a single value for the response in each subject for each fROI for each condition (see Fig. 4).

### 2.6 Does the EMFL identify fROIs and produce selective responses in these fROIs as effectively as standard localizers?

To compare the EMFL with standard localizers—both in their ability to identify selective voxels, and in the selectivity of the response in those voxels—we measured the selectivity of each fROI as a function of (i) whether it was identified with the EMFL or a standard localizer, and (ii) whether its response was measured in the EMFL or a standard localizer. For this analysis, we defined fROIs using a subset of runs from either the EMFL or a standard localizer in individual subjects. Next, we measured the fROI’s response in the remaining held-out runs of both localizers to its preferred and non-preferred conditions (e.g., faces and objects for the FFA). Finally, we tested the significance of contrast (‘preferred’ vs. ‘nonpreferred’), localizer used to define an fROI (EMFL vs. standard), and localizer used to measure its response (EMFL vs. standard) separately for each fROI via a 2 x 2 x 2 ANOVA with these three factors. We also analyzed the entire dataset at once by adding a factor for fROI, resulting in an omnibus 2 x 2 x 2 x 14 ANOVA. These analyses enabled us to test whether the strength of the measured contrast in each fROI depended on the localizer used to identify voxels or the localizer used to measure responses or both, and whether the answer to this question varied across fROIs.

### 2.7 Selectivity of fROIs as a function of fROI volume

The selectivity of an fROI will naturally depend on the volume of the region selected: a hotspot at the center of each individual’s activation is likely to be very selective, but as the volume included in the fROI increases, more voxels with lower selectivity will be included. Thus, an anatomical parcel large enough to accommodate the variable position of fROIs across participants will necessarily include many voxels with weak or no selectivity. (That is why anatomical parcels must be intersected with individual activation maps to adequately define each fROI.) Our next analysis quantifies the dependence of fROI selectivity on fROI volume using a “growing window” method as described in previous work (Kosakowski et al., 2022).

First, we rank-ordered voxels by their significance value for a given contrast in a subset of functional runs. We then computed the average response magnitude to the localizer conditions in held-out runs in a growing selection of voxels, starting from the most selective voxel and gradually increasing the selection size by including less selective voxels, in steps of one voxel at a time. We performed this procedure on individual participants using data from the EMFL (Fig. 6), but also using data across different localizers with the same or similar contrasts. For example, we rank-ordered voxels by the significance of their Faces > Objects contrast during even runs of the EMFL and measured the responses to Faces and Objects conditions, in those same voxels, during odd runs of the FOSS localizer (see Supplementary Fig. S1). This alternative analysis also tests whether our EMFL cross-validates to standard localizers by measuring the response magnitude of conditions in the standard localizer in fROIs defined (as described above) by the EMFL.

### 2.8 Voxel overlap within fROIs measured via dice coefficient

To determine whether EMFL and standard localizers identify similar voxels as belonging to each fROI, we calculated the Dice coefficients between the EMFL and the standard localizers to quantify the degree to which they spatially overlap. First, we defined each fROI separately for each individual, specifically the top 10% of voxels within the relevant parcel based on the relevant functional contrast, separately for the EMFL and standard localizers. We then calculated the Dice coefficient of the overlap both within and between localizers. Dice coefficients range between 0 (meaning no overlap) and 1 (perfect overlap). Dice coefficients within a localizer necessarily required splitting the data into even and odd runs, so for a comparison with equivalent power, we calculated between-localizer Dice coefficients using the same even-odd splits of the data. In addition, to quantify the between-localizer Dice coefficients with maximum power, we also calculated overlap using all runs of the data for each localizer.

## 3 Results

### 3.1 EMFL data reveal the expected activations for both visual and auditory contrasts in most individual participants

First, as an initial reality check on the ability of the EMFL to produce the expected activations, we analyzed the fMRI BOLD responses from each participant with each of the fROI-defining contrasts (see Table 3) using the full set of five runs. Whole-brain activation maps for category-selective contrasts for faces, places, bodies, and words (all compared to objects) are shown for five randomly-selected participants, along with the relevant anatomical constraint parcels, in Figure 2. Visual inspection reveals activations that qualitatively resemble those in the extensive literature on these regions. This finding shows that the simultaneous performance of a relatively demanding task on auditory stimuli does not noticeably compromise the expected visual category-selective activations. Further, for our various contrasts of auditory conditions, whole-brain activation maps for the same five participants reveal the expected activations in most participants as shown in Figure 3. Comparisons of activations for EMFL and standard localizers within the same participants are shown for Theory of Mind and Speech contrasts in Supplementary Figure S2 and for Language in Supplementary Figure S3.

### 3.2 How many participants show each fROI individually?

We next asked how effective EMFL is by asking in how many participants it identifies each fROI using the relatively stringent significance threshold of *p* < 0.001 uncorrected and a requirement of at least 10 voxels in the relevant anatomical parcel for each fROI. By this measure, the EMFL identifies the main fROIs in at least 18 out of 20 participants when all five runs are used (see Table 4); exceptions are the rOFA, rSTS, and VWFA, with fROIs successfully defined by this measure in only 12–14 out of 20 participants (fewer subjects than in the standard localizers for these regions). However, many fMRI studies now define fROIs not using a hard statistical threshold, but instead by choosing the top 10% of most-significant voxels within an anatomical constraint parcel, whether or not these voxels reach a fixed significance level. This method has the advantage of including all participants in an analysis. Indeed, as shown in Supplementary Figure S4, even in participants who did not have 10 voxels teaching the *p* < 0.001 significance level for the weakest fROIs (STS, OFA, and VWFA), most of these participants nonetheless showed the expected selective response profile in held-out data when their fROIs were defined as the top 10% of voxels within the parcel.

**Table 4.** Number of participants (out of 20) who show at least 10 voxels that are significant for the relevant functional contrast (*p* < 0.001) within the relevant anatomical constraint parcel. See Supplementary Table S2 for the average number of significant voxels in each parcel.

	EMFL				Standard localizers			
Runs	odd		all		odd		all	
Hemisphere	left	right	left	right	left	right	left	right
FFA	17	20	18	20	17	18	19	19
OFA	4	8	5	12	9	16	11	20
STS	4	12	2	14	1	16	2	17
PPA	18	18	19	18	16	16	17	17
OPA	17	18	19	20	15	17	19	19
RSC	16	18	18	18	11	15	16	17
EBA	17	18	17	18	18	19	18	20
VWFA	11		14		17		18	
LOC	18	20	20	20	20	19	20	20
RTPJ		8		17		11		17
Speech	20	20	20	20	19	19	20	20
Language network (1)	17		19		8		12	
Language network (2)	19		19		14		18	
Language network (3)	19		20		14		16	
Language network (4)	20		20		17		18	
Language network (5)	20		20		18		19	
Language network (6)	17		18		4		8	
Frontal MD	19	20	20	20	18	16	19	19
Parietal MD	20	19	20	20	16	18	20	20

Contrast significance was computed using either all five runs, or just the odd-numbered runs of either the EMFL or the relevant standard localizer. Empty cells indicate fROIs that are not expected to be robust in the hemisphere indicated (e.g., the VWFA in the right hemisphere).

Can the main fROIs be defined via the hard statistical threshold with even less scan time? Indeed, as shown in Table 4, just three runs of the EMFL (14 minutes of data collection) was still sufficient to identify most fROIs in most participants. Taken together, we conclude that three runs (14 minutes) are sufficient to define most regions in almost all subjects using the hard significance criterion, but five runs are recommended if localization of VWFA, rOFA, or rSTS is important for the study.

### 3.3 The EMFL effectively identifies voxels selectively responsive to the predicted condition(s)

Our primary question is whether the EMFL successfully identifies fROIs that reveal the expected functional selectivities in held-out data. To answer this question, we identified each fROI as the top 10% of voxels within the relevant anatomical parcel in a subset of EMFL runs, and quantified responses using the held-out data from the other EMFL runs, separately for each participant. Figure 4 shows the response to each of the 10 conditions in EMFL in each of 14 well-established fROIs, showing the expected selective responses in each fROI. Results for left hemisphere fROIs are shown in Supplementary Figure S5 and for individual language and MD regions in Supplementary Figure S6. These findings are supported by statistical tests in Supplementary Table S1, which show (with very few exceptions) that the response to the preferred condition was significantly greater than to each of the non-preferred conditions in each fROI.

One exception was the contrast of faces versus bodies, which did not reach significance in the fSTS (*p* = 0.07). This result was expected due to the implied presence of social interaction in videos of bodies, and the role of fSTS in social information processing. Notably, the contrast of visual faces over auditory false belief and false photo stories reached only modest significance in the fSTS (*p* = 0.04 and *p* = 0.005, respectively), perhaps reflecting amodal processing and/or interference in the STS produced by the required mentalization during these stories.

The other exception was the speech region, which did not respond more to speech than to the visual body condition. This result can be explained by the fact that most of the auditory conditions contained speech sounds, and the average response of all of these conditions served as the baseline for the visual responses (because the simultaneous presentation of visual and auditory conditions resulted in the response of each condition in one modality, including the average of all the conditions in the opposite modality). For the same reason, we did not expect the response measured for the irrelevant modality (e.g., the response to auditory stimuli for visual category-selective selective regions) to be zero: each auditory condition occurs simultaneously with visual conditions, and vice versa. The average response across all preferred-modality conditions for each fROI (solid black line in Fig. 4) thus serves as the relevant baseline for the non-preferred modality conditions (dashed black line in Fig. 4) in that fROI.

In sum, as shown in Figure 4, three runs of the EMFL localizer is sufficient to identify each of the standard fROIs, which show the expected selective response profile in the two held-out runs. Supplementary Figure S7 shows that the same is true when the first three runs are used to identify the fROI, and the last two to quantify its response, showing that just three runs suffice to identify fROIs.

### 3.4 Quantifying the similarity of fROIs obtained from EMFL and standard localizers

**Fig. 5.**
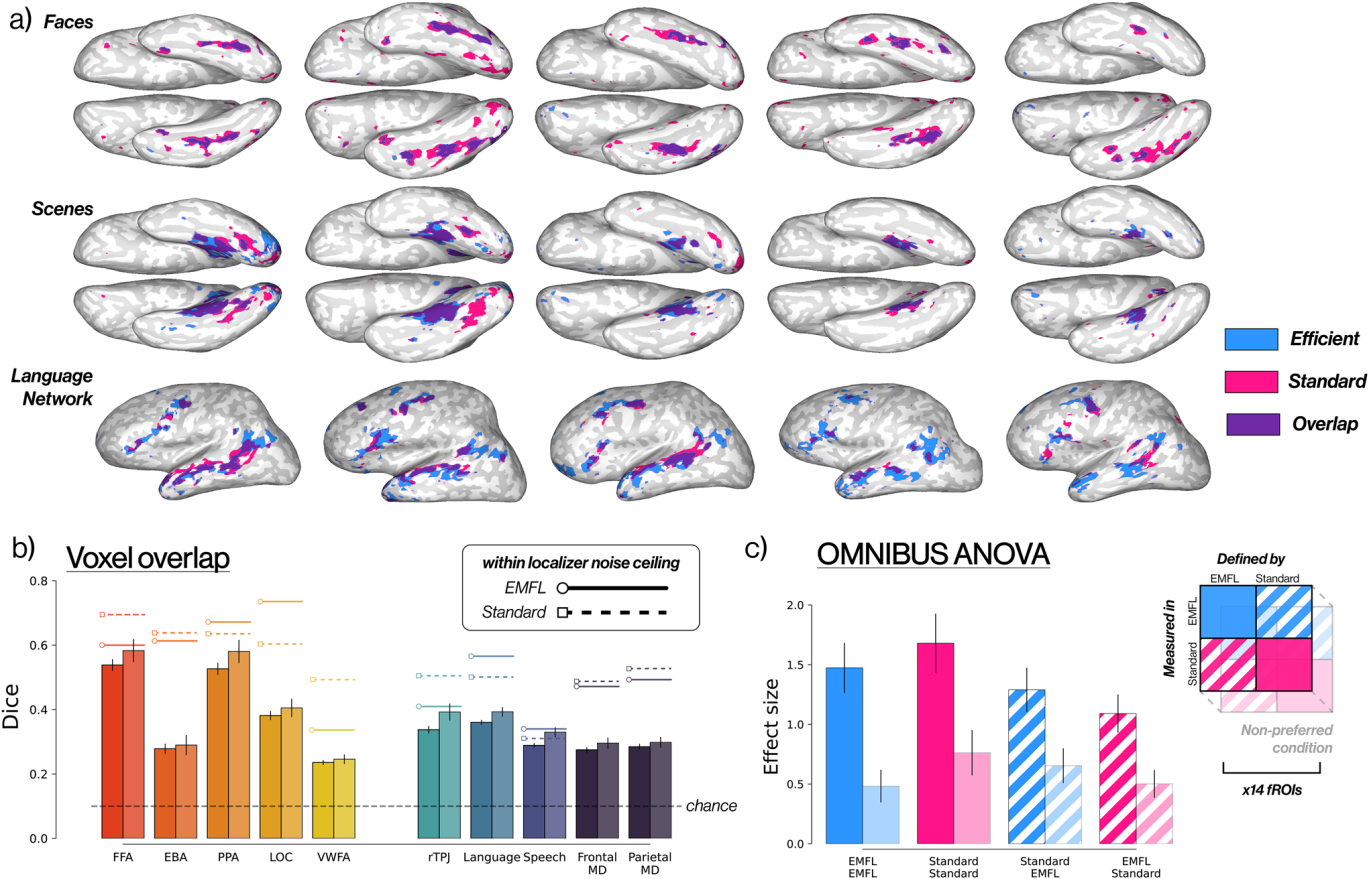
EMFL and standard localizers produce similar brain activity. (a) brain activation maps of three different conditions during EMFL and a standard localizer, plotted in the individual’s native space (same five subjects as Fig. 2). Voxels are colored if they reached a significance threshold of *p* < 0.001 in EMFL (cyan), a standard localizer (magenta), or both (violet) for a given functional contrast. (top) Faces > objects by EMFL and Epstein and Kanwisher (1998), (middle) scenes > objects by EMFL and Epstein and Kanwisher (1998), (bottom) English > non-words in EMFL and sentences > non-words in Fedorenko et al. (2010) (b) Dice coefficient of the top 10% most-selective voxels identified by the EMFL and standard localizers for each region, calculated within an anatomical parcel. Dark bars average across four even-odd splits, light bars use all runs available. Floating bars represent the Dice coefficient within even and odd runs of the same localizer. Dashed line indicates overlap by chance. (c) results of the 2 x 2 x 2 x 14 omnibus ANOVA. Response magnitudes are averaged across fROIs for preferred (bright) versus non-preferred (pale) conditions, measured in the EMFL (blue) or standard (pink) localizer and defined by either the same localizer (solid) or the other localizer (striped). *See Tables 2 and 3 legend for ROI abbreviations.*

Are the same voxels picked out by the EMFL and standard localizers? A qualitative visualization of the activation overlap between EMFL and standard localizers for face, place, and language-selective activity is shown in Figure 5A. To quantify this overlap, we calculated the Dice coefficients between the top 10% most selective voxels for each region identified by the EMFL versus the standard localizer, using all data in each case (Fig. 5B). All Dice coefficients were significantly greater than chance (all *p*-values < 0.001). We then asked whether Dice coefficients were higher within a localizer than between localizers, using equivalent splits of the data within each localizer (see Methods), and found significantly higher overlap between runs of the same localizer compared to the overlap of runs between different localizers.

We also measured the similarity of the relevant stimulus contrast across voxels within the relevant parcel for each fROI via Pearson’s correlation coefficient (Supplementary Fig. S8). The pattern of response across voxels for the localizer contrast within the relevant parcel was significantly correlated between EMFL and standard localizers for all fROIs (all *p*-values < 0.001). However, the correlation between the pattern of response across voxels was significantly higher between splits of the data within a localizer than between localizers for all fROIs, with an exception for the fSTS.

Thus, both Dice and correlation measures show that fROIs identified with EMFL and standard localizers are similar, showing generalization across the many differences between EMFL and the standard localizers (static images vs. movies, presence of stimuli in other modality, etc.).

However, unsurprisingly, the various pairs of localizers do not produce exactly the same pattern of response for each contrast. These differences between localizers remind us that the response of voxels in these regions is not solely determined by the presence versus absence of the preferred stimulus or task.

The lowest similarities between EMFL and standard localizers were found for word and body selective regions. We speculate that this dissimilarity resulted from the dissimilar depiction of word and body stimuli in the EMFL (colored movies of bodies and words on dynamic scrambled background) and the standard localizer (static images of line drawings and text on a white background). Consistent with this interpretation, pilot data from 5 participants who were brought back and scanned on a more standard localizer for body-selective regions, using photographs instead of line drawings, showed a higher Dice coefficient with EMFL than did our previous less-standard localizer based on line drawings.

Similarly, the relatively low correlation between the EMFL Theory of Mind contrast and the “standard” localizer presumably reflects the fact that the standard localizer in this case was a contrast of reading stories about emotional pain versus physical pain, whereas the EMFL localizer uses the classic contrast of false belief > false photo, which isolates similar but not identical mental processes (Jacoby et al., 2016). This nonidentical localizer was used because the standard false belief localizer is less effective when repeated within the same participant.

### 3.5 The selectivity of response does not depend on the localizer used to define a fROI, but does depend (for some fROIs) on which localizer is used to measure responses

To test whether the selectivity of response of each fROI depends on either (i) the localizer used to identify the fROI or (ii) the localizer used to measure responses in that fROI, we performed an omnibus 2 x 2 x 2 x 14 ANOVA across all participants, with factors for contrast (preferred vs. non-preferred condition), localizer used to define the fROI (EMFL vs. standard), localizer used to measure responses (EMFL vs. standard), and parcel (the 14 main functional regions of interest). The results of this ANOVA are shown in Table 5 (see Fig. 5c for summary). The key finding is that the selectivity measured (“contrast”) does not depend on whether the region is defined with EMFL or standard localizers (“definer”), that is, neither the interaction of contrast x definer nor contrast x definer x fROI reached significance. However, for some fROIs, the strength of the contrast measured does depend on whether responses are measured using the EMFL or standard localizers, as reflected in a significant interaction of contrast x measurer x parcel (*p* = 2 ∗ 10^−6^). We, therefore, performed 2 x 2 x 2 ANOVAs separately within each of the 14 fROIs, which are shown in Supplementary Figure S9. These analyses show that although the EMFL and standard localizers are similarly effective for defining all fROIs (the contrast x definer interaction did not reach significance for any of the fROIs), the EMFL produces more selective responses for some fROIs including the PPA and RSC (supported by significant interactions of contrast by measurer, *p* < 0.0001 for each)—likely due to the optimization procedure used to select the particular scene stimuli used in this study (Chen et al., 2024). In only one fROI, the OFA, did the EMFL produce a less selective response than did the standard localizer (interaction of contrast x measurer *p* = 0.02). In sum, these analyses show that the EMFL identifies fROIs as effectively as the standard localizers do, and (with the one exception of the OFA) produces responses in those fROIs that are as selective or more selective than the standard localizers.

**Table 5.** Summary of 2 x 2 x 2 x 14 omnibus ANOVA.

	df	sum_sq	mean_sq	F	PR(>F)
C(contrast)	1	370.0	370.0	990.0	1.5e-178
C(definer)	1	28.0	28.0	76.0	5.4e-18
C(measurer)	1	0.44	0.44	1.2	0.27
C(parcel)	13	580.0	45.0	120.0	2.5e-244
C(contrast):C(definer)	1	0.012	0.012	0.032	0.86
C(contrast):C(measurer)	1	0.33	0.33	0.89	0.35
C(definer):C(measurer)	1	26.0	26.0	69.0	1.4e-16
C(contrast):C(parcel)	13	45.0	3.4	9.3	5.1e-19
C(definer):C(parcel)	13	27.0	2.1	5.6	3.7e-10
C(measurer):C(parcel)	13	170.0	13.0	35.0	2.5e-80
C(contrast):C(definer):C(measurer)	1	18.0	18.0	48.0	6.9e-12
C(contrast):C(definer):C(parcel)	13	1.8	0.14	0.37	0.98
C(contrast):C(measurer):C(parcel)	13	19.0	1.5	4.0	2e-06
C(definer):C(measurer):C(parcel)	13	22.0	1.7	4.6	6e-08
C(contrast):C(definer):C(measurer):C(parcel)	13	8.3	0.64	1.7	0.05
Residual	2100	780.0	0.37		

With main effects for contrast (preferred vs. non-preferred), localizer used to define ROIs (definer, EMFL vs. standard), localizer used to measure responses (measurer, EMFL vs. standard), and fROI (parcel), as well as their interactions.

### 3.6 Characterizing the selectivity of each fROI as a function of fROI volume

**Fig. 6.**
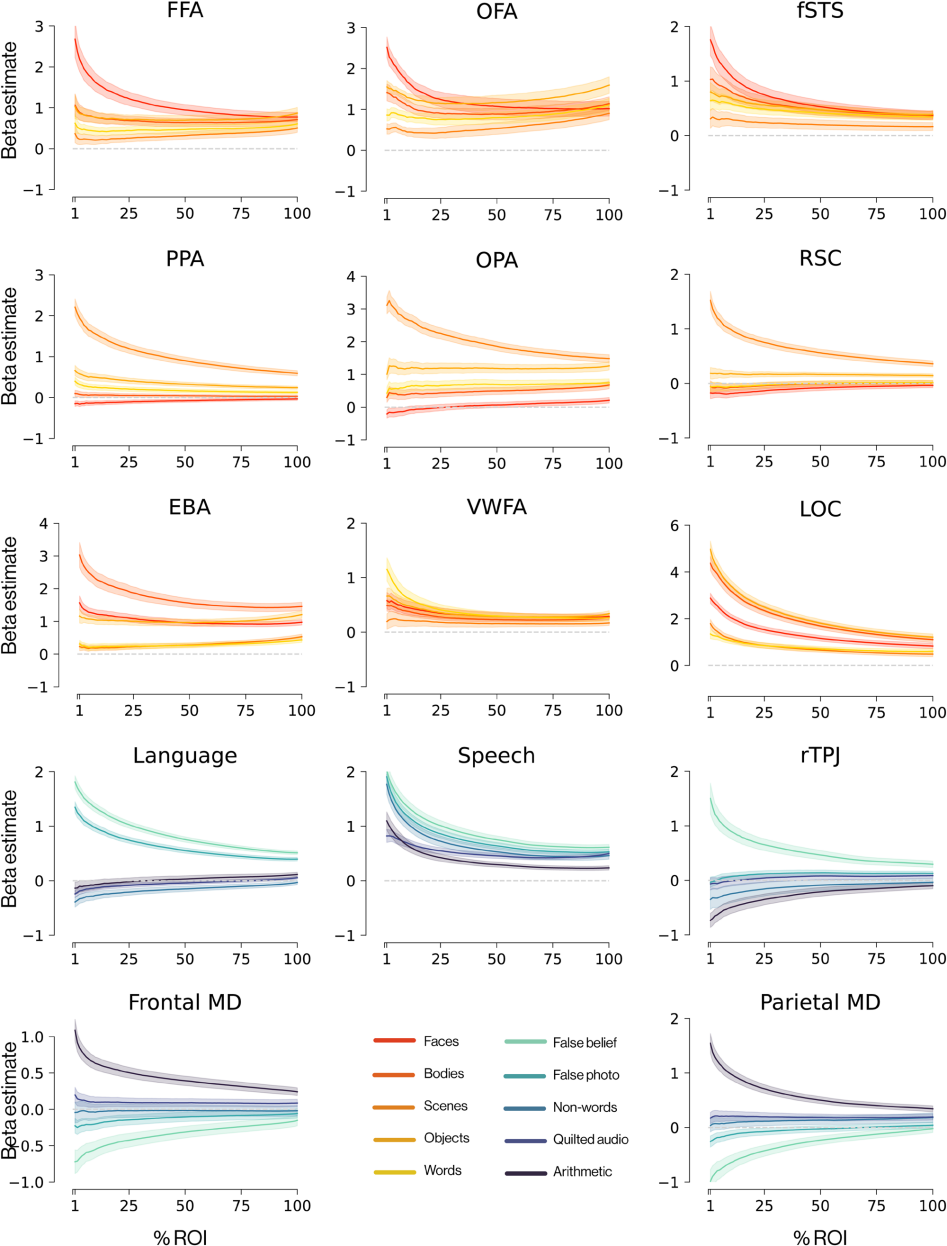
Selectivity of EMFL-defined fROIs decreases as a function of fROI size. Response of each functional region of interest defined by and measured during EMFL. We selected voxels by their functional selectivity during the first three runs and measured their beta estimates during the final two held-out runs. Response magnitudes (y-axis) are plotted as a function of fROI size (x-axis, % of voxels in the parcel that are included in the fROI).

The ultimate test of a functional localizer is its ability to identify voxels that produce the intended selectivity in held-out data. However, the exact number of voxels which reach a fixed significance threshold for a given contrast can vary considerably across participants. As noted above, a standard method for addressing this problem is to define the fROIs as the top 10% most-significant voxels within a contrast-relevant constraint parcel, regardless of whether those voxels meet a fixed significance threshold. Here, we provide support for that approach by showing how the selectivity in held-out data decreases as less-significant voxels are included in the fROI. We quantify in Figure 6 this drop-off in selectivity of each fROI as the volume of that fROI increases. As expected, fROIs show their strongest selectivity in the top 10% of voxels, and most fROIs still show a twofold response to their preferred condition compared to any non-preferred condition, though a few show more modest patterns of selectivity. For all fROIs, if 100% of the parcel is included then selectivity is weak (which is why functional localizers are needed!). In a similar analysis, we further cross-validate the selectivity of each fROI by using the EMFL to define voxels and then measure their responses in a standard localizer (Supplementary Fig. S1).

## 4 Discussion

The discovery that the human brain contains a set of cortical regions with distinctive functional response profiles, each present in virtually every neurotypical individual, invites research into the developmental and evolutionary origins, as well as the representations, computations and behavioral relevance of these regions. Yet the individual variability in the precise anatomical location of these regions means that they can only be studied precisely if they are first functionally defined in each participant individually. Here, we present and evaluate an efficient functional localizer that reliably identifies, in just 14 minutes of fMRI scan time, brain regions in each individual that are selectively engaged in the visual processing of faces, scenes, bodies, objects, and words, as well as regions engaged in speech perception, language understanding, theory of mind, and domain-general cognitive demand. We further cross-validate this localizer with the corresponding established localizers by showing substantial topographic overlap and similar expected response selectivity in held-out data. We find that the selectivity of these regions in held-out data is as strong when they are defined using our new efficient multifunction localizer (EMFL) as they are when defined by standard established localizers. The EMFL is available on GitHub (see Methods for the repository link) and should be widely applicable in human cognitive neuroscience research, enabling multiple regions to be studied in each experiment, and making possible a cumulative research program in which studies across participants and labs can refer to the same regions.

Our results further reinforce the functional selectivity of each region, not just with respect to the standard conditions it is often contrasted with (e.g., faces vs. objects for the FFA), but with respect to a broader set of conditions spanning vision, hearing, social cognition, and domain-general demand. Indeed, almost all of our fROIs showed a significantly higher response to their preferred condition than to each of the other non-preferred conditions (One exception was the fSTS, for which the higher response to visually presented faces over bodies did not reach significance, and speech regions, which did not respond more to speech than some of the visual conditions presumably because speech sounds are present in almost all visual blocks). While the signature response profiles of these regions have long been widely reported (and in some cases, extended to animal physiology work; e.g., Tsao et al., 2006), recent discussion oddly seems to question the existence or significance of functional specificity in the brain (e.g., Pessoa, 2023; Westlin et al., 2023). Our data, paradigm and analysis tools are publicly available, enabling anyone to easily replicate these findings for themselves.

Beyond demonstrating the efficacy of an efficient localizer and showing the functional selectivity of the regions, it identifies across a broad swath of stimuli and tasks, several findings from our study are notable. First, we found strong selectivity for visual conditions even when visual stimuli were task-irrelevant and participants were engaged in an unrelated and demanding auditory task. Although attentional modulation of category-selective regions is well established (e.g., O’Craven et al., 1999; Wojciulik et al., 1998), few if any prior studies have shown as we do here that strongly selective responses, nonetheless, remain for unattended visual stimuli. Additional pilot data (shown in Supplementary Fig. S10) from five participants who were brought back to perform the EMFL experiment without the concurrent auditory stimuli or tasks showed no clear increase in selectivity of category-selective regions (see also Bugatus et al., 2017; Harel et al., 2014; Mur et al., 2025). Second, the face-selective region in the STS did not respond significantly to speech sounds, in contrast to prior reports (Deen et al., 2015). However, task-relevant mentalization produced by auditory false belief and false photo stories modulated their contrast significance against visually presented faces. Conversely speech-selective regions did not respond more to faces than other stimuli, suggesting a segregation of these two responses in the STS. Third, the multiple demand regions responded strongly to auditory arithmetic but barely above a fixation baseline for the other auditory contrasts, including speech perception, language understanding, and theory of mind, even though these tasks were at least as difficult as the auditory arithmetic task (Fig. 1D). This finding shows that although multiple demand regions respond across a wide range of cognitive tasks, this does not include speech perception, language understanding, or theory of mind (Fedorenko & Shain, 2021; Saxe et al., 2006).

Of course, functionally distinct cortical regions do not act alone, but receive input from, send outputs to, and presumably interact online with numerous other areas. Indeed, analyses of the correlations of the fMRI signal at rest (or during task performance) have identified “functional networks” spanning many regions. Although these fMRI correlations between regions do not necessarily reflect structural connectivity, they do reliably identify groups of regions that apparently work together (Yeo et al., 2011). This macroscopic level of analysis of brain-wide networks is complementary to and synergistic with more fine-grained analyses of individual cortical regions; both levels of analysis will be important for understanding brain function. Further, resting functional correlation analyses have also been shown to allow efficient functional localization of some functionally distinct brain regions, as demonstrated by cross-validation with standard task-based functional localizers (Braga et al., 2020; Du et al., 2024). Language regions can even be accurately identified from functional correlation analyses of single runs of resting state data (Du et al., 2025; Shain & Fedorenko, 2025).

Other studies have proposed efficient methods for localizing functional regions using task and movie data. Lee et al. (2024) demonstrated the ability to localize language regions in just 3.5 minutes of scan time while maintaining the fidelity of its spatial layout and functional response profile. Tuckute et al. (2024) found similar results with their 3.5 minute localizer based on speeded reading, which additionally showed a functional dissociation of the language network from the topographically-nearby multiple demand network. The hyperalignment method introduced by Haxby et al. (2011) can also be used to identify functionally specific regions from fMRI data while participants watch movies (Jiahui et al., 2020). Finally, one recent study found that many high-level functionally specific visual areas could be accurately identified from a 10-minute resting functional scan (Molloy et al., 2024). While all of these methods are useful, our functional localizer is the only one with broad coverage, identifying the mostly widely studied perceptual and cognitive regions, in as little as 14 minutes of scan time. As such, we hope to encourage researchers to include this localizer in a wide range of fMRI studies, enabling them to complement other planned analyses with a quantification of their effects within the main established fROIs.

Future research should compare the efficacy of EMFL with other methods such as individual-subject rsfMRI methods (Braga et al., 2020; Du et al., 2025; Shain & Fedorenko, 2025), connectivity fingerprints (Molloy et al., 2024), group-based fROIs (see Fig. 2D, also Saxe et al., 2006), multimodal anatomical parcels (Glasser et al., 2016), and movie-based hyperalignment (Jiahui et al., 2020).

Despite its advantages, our localizer has some limitations. First, the *‘Does this come next?’* task feels at first counterintuitive and demanding, as participants must perform an auditory task while salient and distracting but irrelevant visual movies are presented. However, most participants quickly realized that they can, in fact, successfully selectively attend to the auditory stimuli, performing above chance in every condition. While very young, very old, or cognitively impaired individuals may not be able to perform our task adequately, our pilot data from three high-functioning elderly participants indicate that they had no trouble with the paradigm, and strong activations were obtained. A second limitation is that our localizer is less effective for identifying some fROIs like the VWFA and OFA, so studies focusing on these regions may wish to use localizers optimized for identifying these regions. Nonetheless, these regions—when defined by the EMFL—show similar profiles of selectivity as those found in recent studies (Li et al., 2024). Finally, EMFL does not localize all cortical regions implicated in distinct mental functions. Functional contrasts not targeted in this localizer include visual perception of color (Lafer-Sousa et al., 2016), motion (Tootell et al., 1995), hands (Bracci et al., 2010), tools (Bracci et al., 2012), and third-party social interactions (Isik et al., 2017); auditory perception of music (Norman-Haignere et al., 2015) or pitch (Norman-Haignere et al., 2013); and motor functions like reaching, grasping, and eye movements (Gallivan & Culham, 2015), episodic projection (DiNicola et al., 2020), and intuitive physical reasoning (Fischer et al., 2016). Note, however, that because we provide the code and stimuli for the experiment, it would be straightforward to sub in alternate stimulus conditions or tasks to localize other functional regions of interest.

In sum, we show here that 14 of the most widely studied function regions of interest can be functionally localized in each participant individually in as little as 14 minutes of scan time. Our method cross-validates with standard localizers for individual functional contrasts, and shows selectivity in held-out data as strong as found for standard localizers that take four times as long to run. Our tasks, stimuli, and analysis methods are available online, so anyone can easily add this short localizer to an existing scanning protocol, enabling them to test how these established fROIs respond in their paradigm. In this fashion, we hope to help build a cumulative research program across participants, methods, and labs.

## Supplementary Material

Supplementary Material

## Data Availability

Scripts for the EMFL and relevant analyses are available at these GitHub repositories: https://github.com/aimarvi/efficient_localizer/ (localizer) and https://github.com/aimarvi/emfl_analysis (analysis). fMRI data are available on OpenNeuro: https://openneuro.org/datasets/ds006179/versions/1.0.1.
