# Supplementary material for: An efficient multifunction fMRI localizer for high-level visual, auditory, and cognitive regions in humans
Source: Imaging Neurosci (Camb). 2025 Oct 14;3:IMAG.a.905. doi: 10.1162/IMAG.a.905 (PMC12521984; doi:10.1162/IMAG.a.905)
Supplement: Supplementary Material [file IMAG.a.905_supp.pdf]

Supplemental Material: An Efficient Multifunction fMRI Localizer for High-Level Visual, Auditory, and Cognitive Regions in Humans

September 9, 2025

Supplementary content

	**Fa**	**B**	**S**	**O**	**W**	**FB**	**FP**	**NW**	**QLT**	**MATH**
**FFA**		3e-07	3e-08	1e-08	1e-07	2e-07	3e-07	5e-08	8e-08	1e-08
**OFA**		2e-05	1e-07	0.0001	8e-06	3e-06	9e-06	7e-06	2e-05	6e-07
**fSTS**		0.07	0.0002	0.002	0.0003	0.04	0.005	0.0002	2e-06	0.0006
**PPA**	1e-10	1e-10		1e-09	4e-10	7e-10	6e-10	2e-10	5e-10	2e-10
**OPA**	3e-12	6e-13		1e-10	1e-11	7e-12	6e-12	7e-12	2e-11	2e-12
**RSC**	7e-11	3e-09		2e-08	6e-10	8e-09	1e-09	5e-09	3e-09	4e-09
**EBA**	2e-08		1e-10	4e-08	5e-10	3e-08	4e-08	9e-10	7e-10	2e-10
**VWFA**	0.0003	0.0003	2e-06	0.0003		7e-06	0.0001	7e-07	6e-05	0.009
**LOC**	2e-07	0.4	2e-12		5e-12	2e-10	2e-08	1e-10	2e-09	2e-10
**Language**	6e-09	1e-09	6e-10	3e-07	2e-09			2e-10	1e-08	2e-08
**Speech**	2e-05	0.4	0.0006	0.007	4e-05	1.0	0.6		8e-07	2e-07
**rTPJ**	2e-09	3e-08	4e-07	4e-09	3e-09		4e-09	2e-10	2e-07	3e-09
**Frontal MD**	1e-08	4e-10	4e-10	2e-09	2e-08	6e-10	6e-09	3e-09	1e-09	
**Parietal MD**	1e-08	7e-09	2e-07	3e-08	1e-07	5e-09	6e-08	3e-08	2e-08	

Supp. Table 1: **Contrast significance across all stimulus conditions.** Significance p values of one-tailed paired t-tests performed within each fROI, comparing the preferred condition to each of the non-preferred conditions. Blank cells indicate preferred conditions. *n.b. several contrasts are not predicted to be significant such as Objects > Bodies or Objects > Faces for LOC and Non-words > False Belief or Non-words > False Photo for rTPJ.*

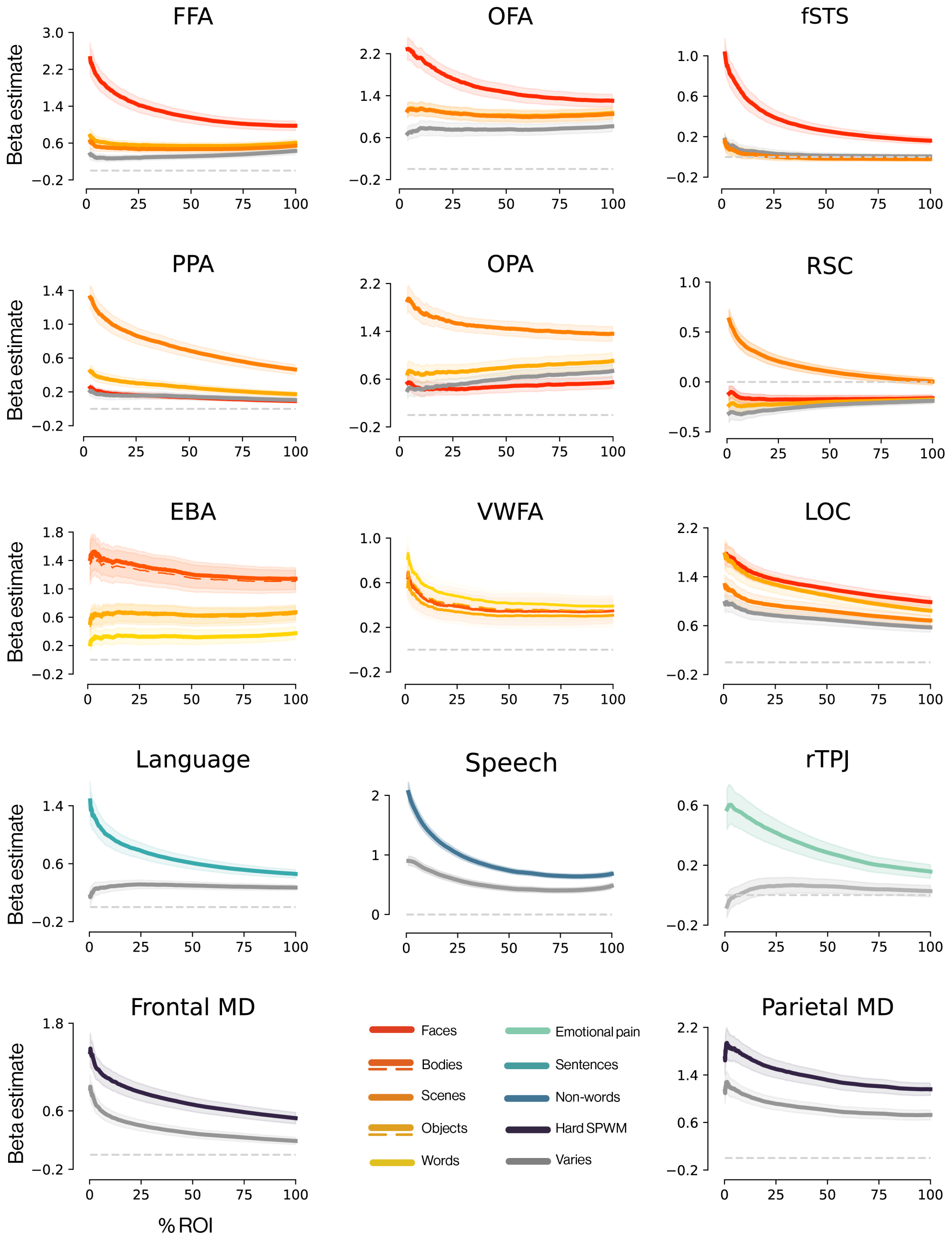

Supp. Figure 1: **Selectivity of EMFL-defined fROIs cross-validates to standard localizers.** Response of each functional region of interest defined by EMFL. We selected voxels by their functional selectivity during a subset of runs and measured their effect size during held-out runs of the relevant standard localizer. Selectivity (y-axis) is plotted as a function of ROI size (x-axis) by averaging the effect size of increasingly larger voxel clusters. *SPWM: spatial working memory, see Tables* **??**, **??** *legend for ROI abbreviations. Dashed curves represent line-drawn versions of the same stimuli, and grey curves represent non-preferred conditions: scrambled objects (visual), non-words (language), physical pain (rTPJ), degraded audio (speech), easy SPWM (MD).*

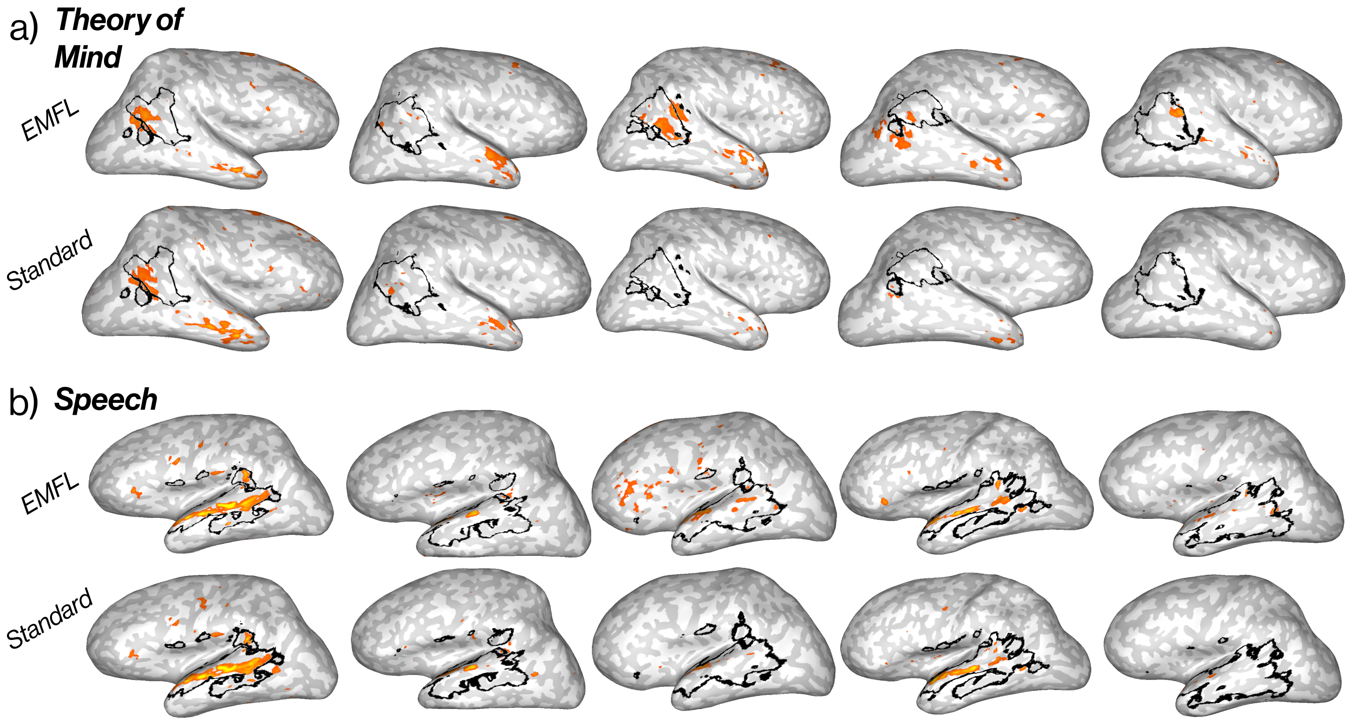

Supp. Figure 2: **Comparison of brain activation maps across EMFL and a standard localizer.** Each brain shows the functional contrast for (a) theory of mind and (b) speech regions as $-log(p\text{-}value) * sgn(t\text{-}test)$, thresholded above +3. Surface projections of relevant anatomical parcels are outlined in black.

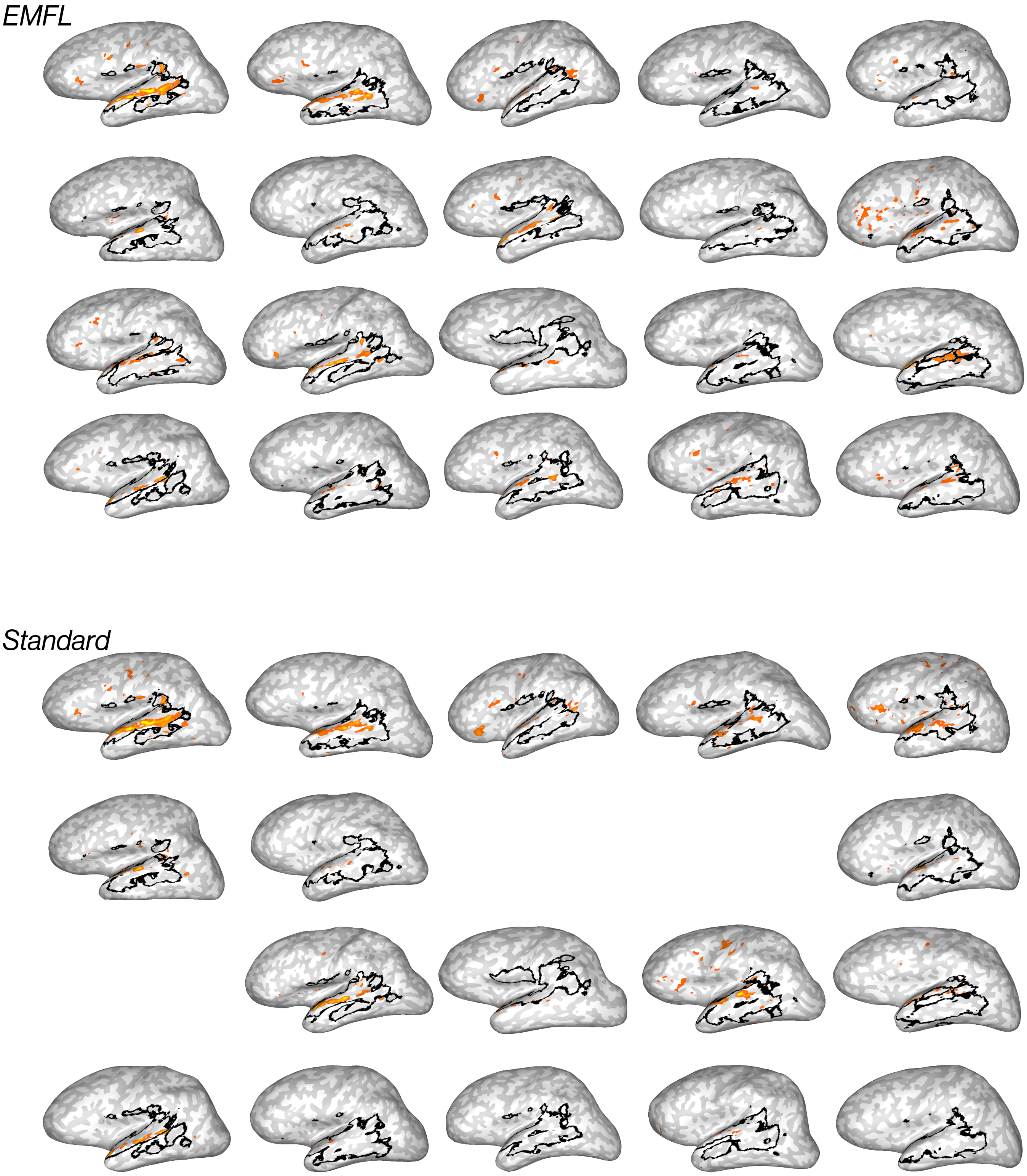

Supp. Figure 3: **Left hemisphere speech activations in all 20 subjects.** Each brain shows the functional contrast for a given condition as $-log(p\text{-}value) * sgn(t\text{-}test)$, thresholded above $+3$. Surface projections of relevant anatomical parcels are outlined in black. Three subjects did not perform the standard speech localizer.

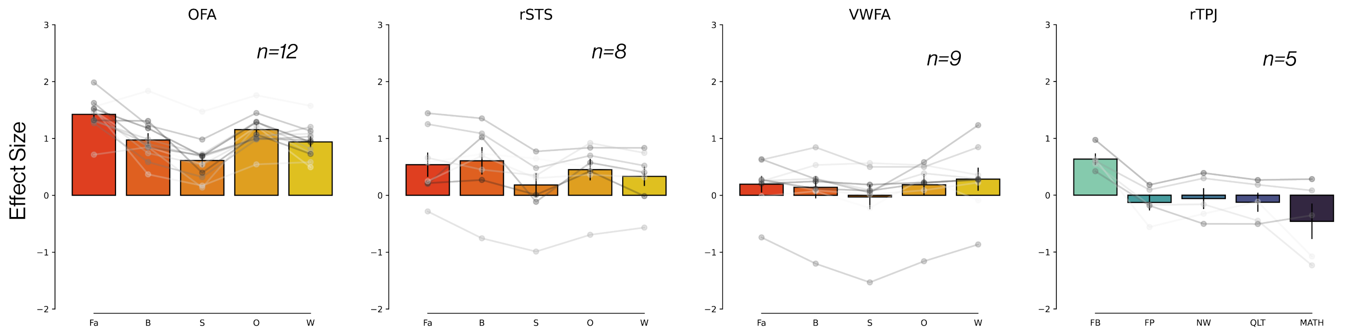

Supp. Figure 4: **Measured selectivity in subjects with <10 significant voxels.** Average effect size of the top 10% of voxels sorted by contrast selectivity. Each plot contains data only from subjects who did not reach the 10-voxel threshold as measured in Table **??**. Beta weights from individual subjects are plotted as gray dots and connected with a line.

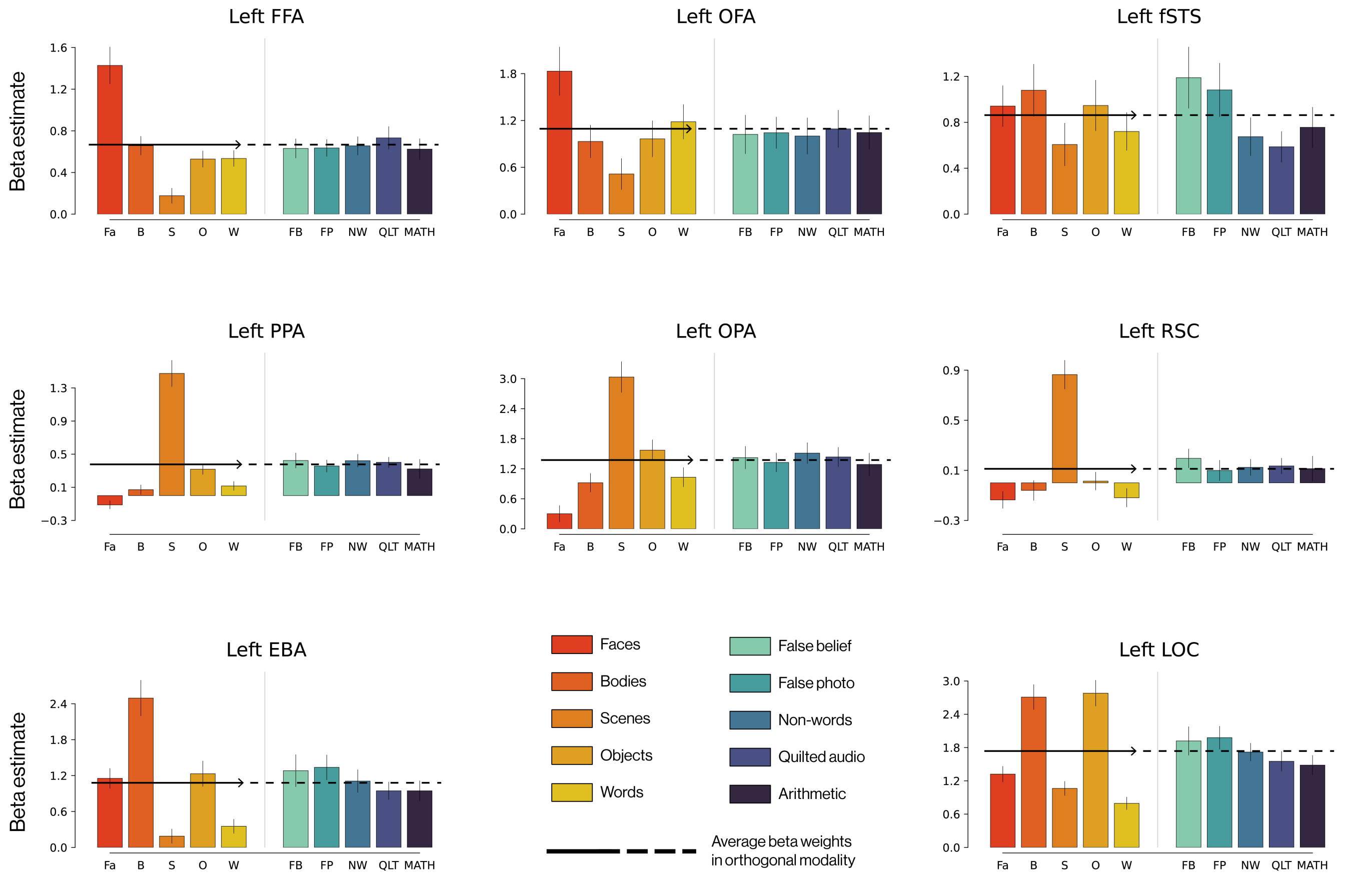

Supp. Figure 5: **Left hemisphere EMFL selectivity.** Beta estimates in held-out runs of eight left hemisphere fROIs in the ventral visual stream.

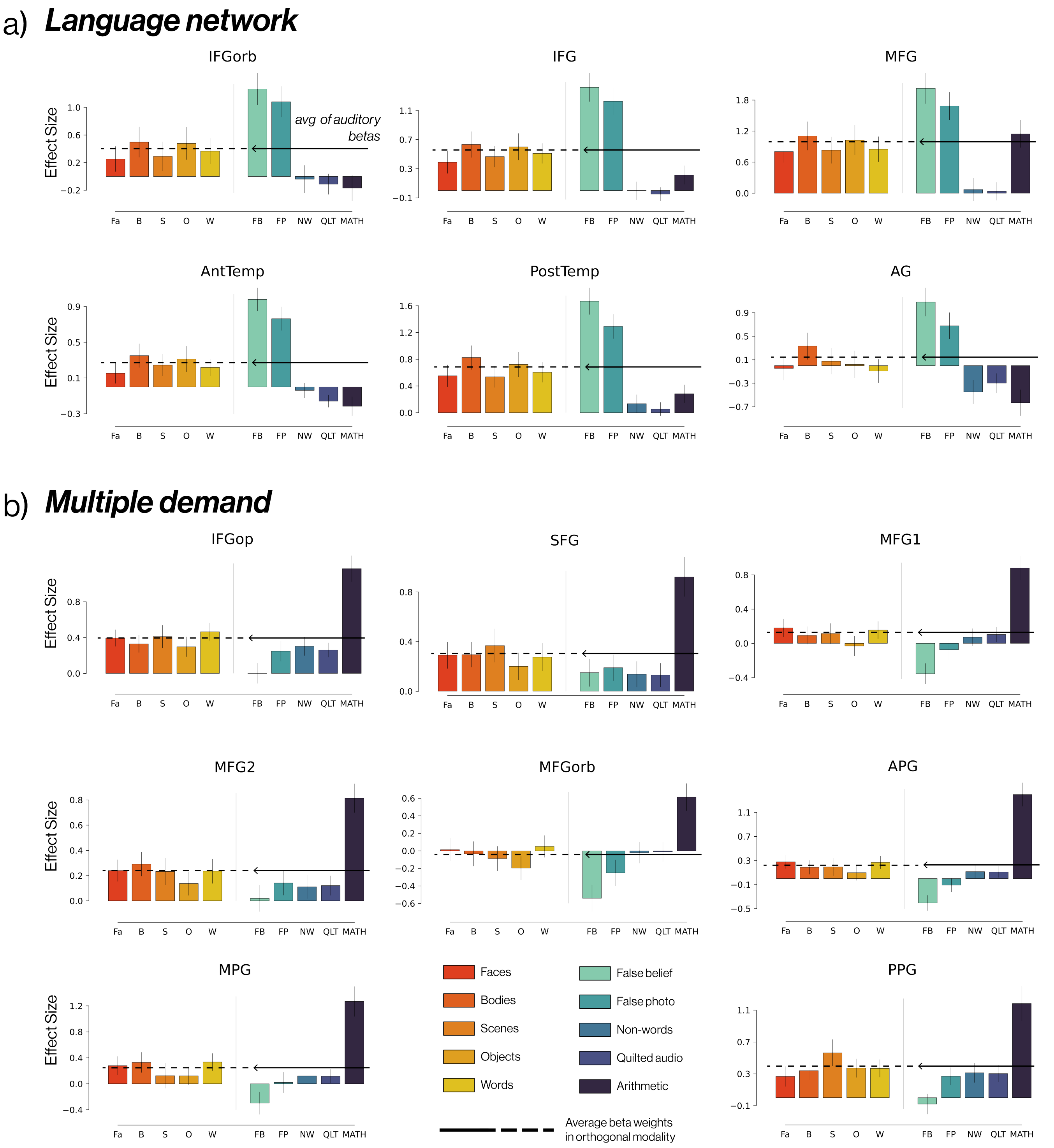

Supp. Figure 6: **Responses of Individual Language and MD Regions.** Response of individual functional regions of interest defined by EMFL to each of the ten conditions in EMFL, performed within (a) the left hemisphere language network and (b) the bilateral multiple demand network. *See Table* **??**, **??** *for ROI abbreviations.*

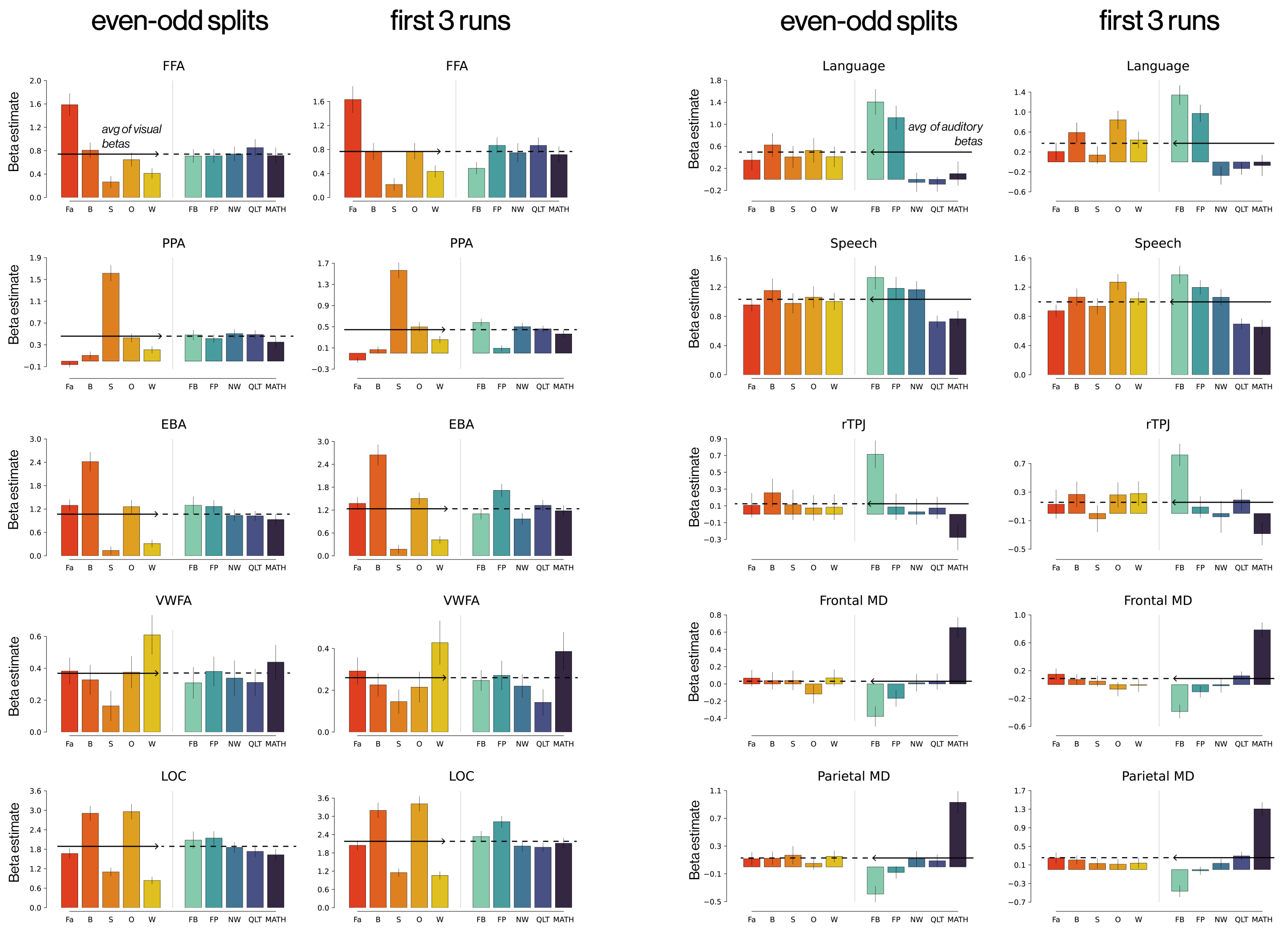

Supp. Figure 7: **fROIs identified with first three runs show comparable selectivity.** Beta estimates for each stimulus condition in held-out runs of the EMFL. 'even-odd splits' averages response measurements in held-out data across even- and odd-numbered runs. 'first 3 runs' uses the first three runs (i.e. 14 minutes) to identify fROIs, and measures responses in the final two held-out runs.

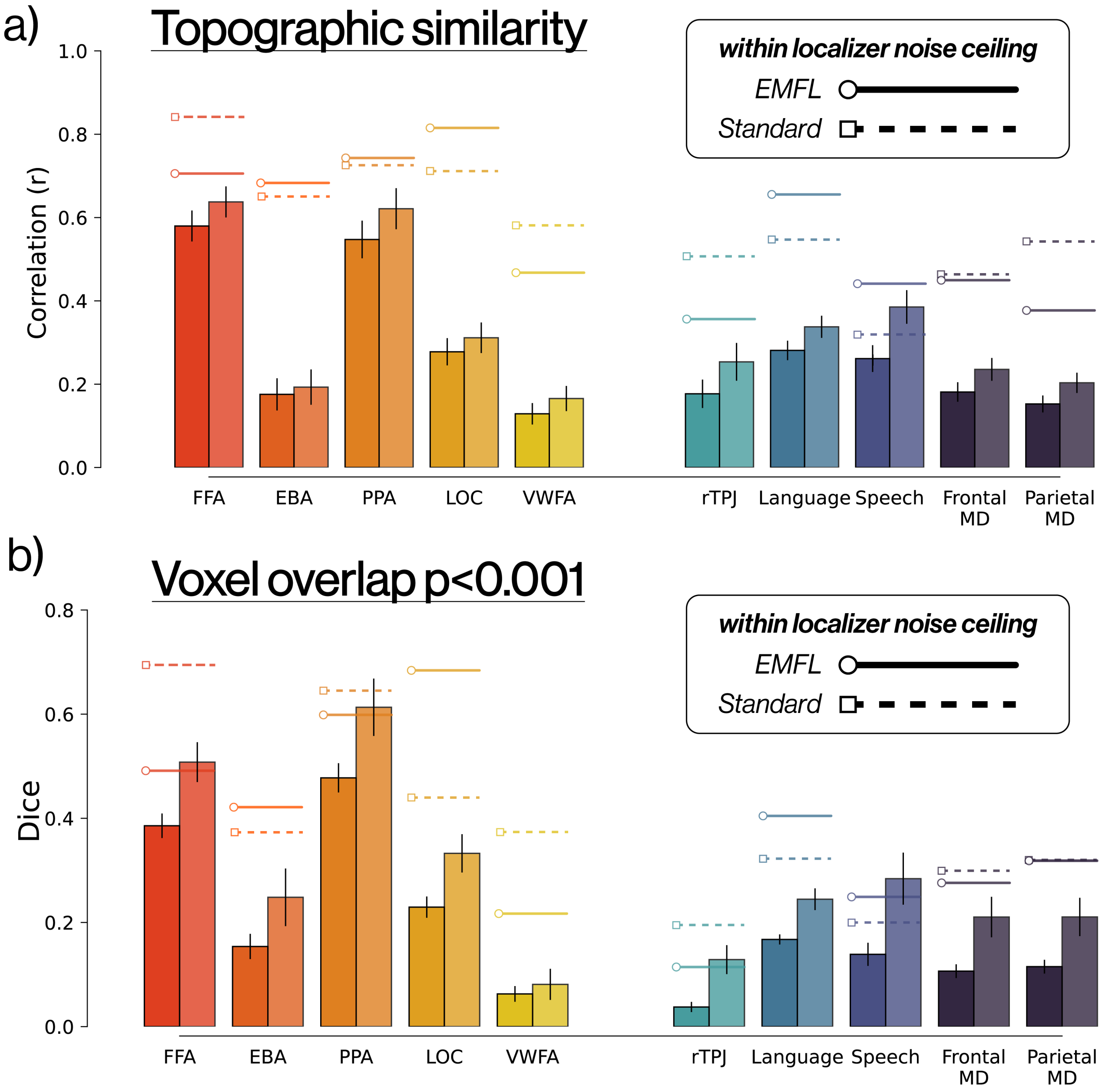

Supp. Figure 8: **Alternative measures of similarity between EMFL and standard localizers within an anatomical parcel.**

(a) Correlation of population-level voxel responses, measured via Pearson's r. (b) Dice coefficient of voxels passing a significance threshold of $p < 0.001$. Dark bars average across four even-odd splits, light bars use all runs available. Solid and dashed lines represent within-localizer split-half noise ceilings.

	EMFL				Standard localizers			
**Runs**	odd		all		odd		all	
**Hemisphere**	left	right	left	right	left	right	left	right
**FFA**	30 / 318	57 / 724	39 / 318	77 / 724	49 / 318	120 / 724	71 / 318	157 / 724
**OFA**	5 / 100	14 / 439	8 / 100	17 / 439	13 / 100	34 / 439	17 / 100	50 / 439
**fSTS**	8 / 152	44 / 929	3 / 152	50 / 929	2 / 152	38 / 929	4 / 152	69 / 929
**PPA**	95 / 412	121 / 400	117 / 412	146 / 400	50 / 412	73 / 400	81 / 412	111 / 400
**OPA**	40 / 369	68 / 417	55 / 369	86 / 417	28 / 369	48 / 417	57 / 369	82 / 417
**RSC**	48 / 350	79 / 498	63 / 350	103 / 498	23 / 350	45 / 498	45 / 350	78 / 498
**EBA**	109 / 839	135 / 1135	134 / 839	167 / 1135	77 / 850	192 / 1150	105 / 850	240 / 1150
**VWFA**	18 / 1238		26 / 1238		43 / 1254		66 / 1254	
**LOC**	338 / 2644	354 / 2248	588 / 2644	590 / 2248	254 / 2644	222 / 2248	383 / 2644	314 / 2248
**rTPJ**		90 / 3501		132 / 3501		73 / 3501		156 / 3501
**Speech**	298 / 12571	298 / 12571	398 / 12571	398 / 12571	270 / 12442	270 / 12442	509 / 12442	509 / 12442
**Language (1)**	62 / 480		106 / 480		28 / 480		46 / 480	
**Language (2)**	125 / 895		197 / 895		55 / 895		91 / 895	
**Language (3)**	118 / 576		157 / 576		59 / 576		83 / 576	
**Language (4)**	235 / 1821		343 / 1821		158 / 1821		235 / 1821	
**Language (5)**	447 / 3073		614 / 3073		229 / 3073		332 / 3073	
**Language (6)**	80 / 767		130 / 767		17 / 767		23 / 767	
**Frontal MD**	398 / 5192	372 / 4972	655 / 5192	692 / 4972	287 / 5192	352 / 4972	640 / 5192	857 / 4972
**Parietal MD**	602 / 5935	366 / 5810	912 / 5935	627 / 5810	387 / 5935	447 / 5810	1011 / 5935	1183 / 5810

Supp. Table 2: **Average number of significant voxels in each anatomical parcel.** Number of voxels that reach a significance value of $p < 0.001$ in each anatomical parcel for the relevant functional contrast. p-values were calculated using either all runs or only odd-numbered runs in either the EMFL or a standard localizer. Each cell represents an average across individual subjects, relative to the total size of each parcel. *n.b. the same parcel may differ slightly in total volume across EMFL and standard localizers because these scans were run in different scanning sessions, and alignment of the parcel to anatomicals was conducted separately for each session, resulting in small differences in parcel size.*

FFA	df	sum_sq	mean_sq	F	PR(>F)
**C(contrast)**	1.0	44.0	44.0	80.0	1.1e-15
**C(definer)**	1.0	3.6	3.6	6.6	0.011
**C(measurer)**	1.0	0.55	0.55	0.99	0.32
**C(contrast):C(definer)**	1.0	0.053	0.053	0.097	0.76
**C(contrast):C(measurer)**	1.0	1.1	1.1	1.9	0.17
**C(definer):C(measurer)**	1.0	0.69	0.69	1.2	0.27
**C(contrast):C(definer):C(measurer)**	1.0	0.31	0.31	0.56	0.46
**Residual**	152.0	84.0	0.55		

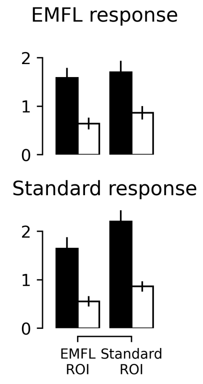

OFA	df	sum_sq	mean_sq	F	PR(>F)
**C(contrast)**	1.0	20.0	20.0	49.0	6.4e-11
**C(definer)**	1.0	6.5	6.5	16.0	8.1e-05
**C(measurer)**	1.0	0.015	0.015	0.037	0.85
**C(contrast):C(definer)**	1.0	0.049	0.049	0.12	0.73
**C(contrast):C(measurer)**	1.0	2.1	2.1	5.3	0.023
**C(definer):C(measurer)**	1.0	0.021	0.021	0.052	0.82
**C(contrast):C(definer):C(measurer)**	1.0	0.54	0.54	1.4	0.25
**Residual**	152.0	60.0	0.4		

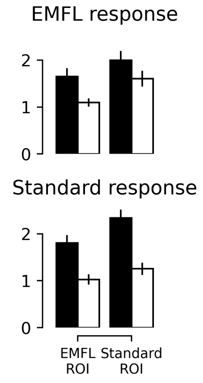

fSTS	df	sum_sq	mean_sq	F	PR(>F)
**C(contrast)**	1.0	10.0	10.0	44.0	6e-10
**C(definer)**	1.0	0.28	0.28	1.2	0.27
**C(measurer)**	1.0	7.1	7.1	31.0	1.2e-07
**C(contrast):C(definer)**	1.0	0.079	0.079	0.35	0.56
**C(contrast):C(measurer)**	1.0	0.019	0.019	0.084	0.77
**C(definer):C(measurer)**	1.0	0.63	0.63	2.7	0.1
**C(contrast):C(definer):C(measurer)**	1.0	0.07	0.07	0.3	0.58
**Residual**	152.0	35.0	0.23		

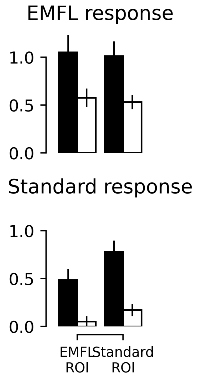

Supp. Figure 9: **ANOVA results and ROI-level plots for individual regions.** Bars represent beta estimates for preferred (black) and non-preferred (white, averaged across all non-preferred) conditions.

PPA	df	sum_sq	mean_sq	F	PR(>F)
**C(contrast)**	1.0	33.0	33.0	130.0	1.2e-22
**C(definer)**	1.0	0.15	0.15	0.6	0.44
**C(measurer)**	1.0	2.7	2.7	11.0	0.0012
**C(contrast):C(definer)**	1.0	0.018	0.018	0.07	0.79
**C(contrast):C(measurer)**	1.0	1.1	1.1	4.5	0.035
**C(definer):C(measurer)**	1.0	0.38	0.38	1.5	0.22
**C(contrast):C(definer):C(measurer)**	1.0	0.29	0.29	1.2	0.28
**Residual**	152.0	38.0	0.25		

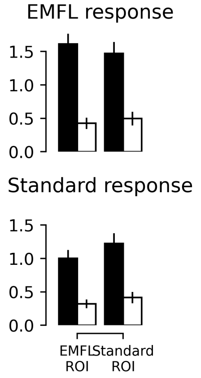

OPA	df	sum_sq	mean_sq	F	PR(>F)
**C(contrast)**	1.0	63.0	63.0	100.0	1.2e-18
**C(definer)**	1.0	4.2	4.2	6.9	0.0097
**C(measurer)**	1.0	6.6	6.6	11.0	0.0013
**C(contrast):C(definer)**	1.0	0.045	0.045	0.072	0.79
**C(contrast):C(measurer)**	1.0	1.1	1.1	1.9	0.17
**C(definer):C(measurer)**	1.0	3.2	3.2	5.2	0.024
**C(contrast):C(definer):C(measurer)**	1.0	0.89	0.89	1.4	0.23
**Residual**	152.0	93.0	0.61		

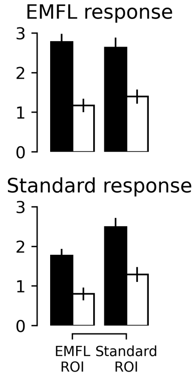

RSC	df	sum_sq	mean_sq	F	PR(>F)
**C(contrast)**	1.0	22.0	22.0	230.0	4.6e-32
**C(definer)**	1.0	0.0018	0.0018	0.019	0.89
**C(measurer)**	1.0	6.1	6.1	64.0	2.8e-13
**C(contrast):C(definer)**	1.0	1e-05	1e-05	0.00011	0.99
**C(contrast):C(measurer)**	1.0	0.68	0.68	7.2	0.0081
**C(definer):C(measurer)**	1.0	0.18	0.18	1.9	0.17
**C(contrast):C(definer):C(measurer)**	1.0	0.095	0.095	1.0	0.32
**Residual**	152.0	14.0	0.095		

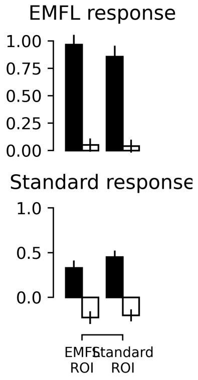

Supp. Figure 9-2: **ANOVA results and ROI-level plots for individual regions.** Bars represent beta estimates for preferred (black) and non-preferred (white, averaged across all non-preferred) conditions.

EBA	df	sum_sq	mean_sq	F	PR(>F)
**C(contrast)**	1.0	38.0	38.0	52.0	2.1e-11
**C(definer)**	1.0	12.0	12.0	16.0	8.6e-05
**C(measurer)**	1.0	8.1	8.1	11.0	0.0011
**C(contrast):C(definer)**	1.0	0.094	0.094	0.13	0.72
**C(contrast):C(measurer)**	1.0	2.0	2.0	2.8	0.094
**C(definer):C(measurer)**	1.0	7.5	7.5	10.0	0.0015
**C(contrast):C(definer):C(measurer)**	1.0	8.5	8.5	12.0	0.00079
**Residual**	152.0	110.0	0.72		

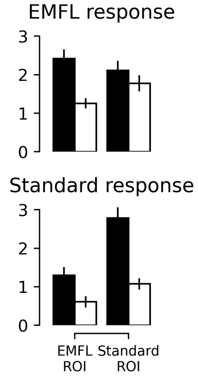

VWFA	df	sum_sq	mean_sq	F	PR(>F)
**C(contrast)**	1.0	2.5	2.5	10.0	0.0019
**C(definer)**	1.0	0.27	0.27	1.1	0.3
**C(measurer)**	1.0	3.4	3.4	13.0	0.00036
**C(contrast):C(definer)**	1.0	0.29	0.29	1.1	0.29
**C(contrast):C(measurer)**	1.0	0.52	0.52	2.1	0.15
**C(definer):C(measurer)**	1.0	1.7	1.7	6.8	0.01
**C(contrast):C(definer):C(measurer)**	1.0	1.3	1.3	5.2	0.024
**Residual**	152.0	38.0	0.25		

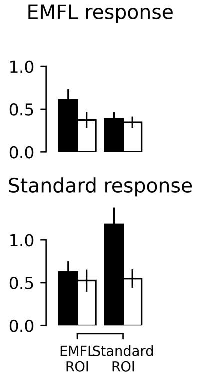

LOC	df	sum_sq	mean_sq	F	PR(>F)
**C(contrast)**	1.0	70.0	70.0	140.0	4.8e-23
**C(definer)**	1.0	0.16	0.16	0.32	0.57
**C(measurer)**	1.0	8.5	8.5	17.0	6.7e-05
**C(contrast):C(definer)**	1.0	0.78	0.78	1.5	0.22
**C(contrast):C(measurer)**	1.0	8.3	8.3	16.0	8.3e-05
**C(definer):C(measurer)**	1.0	6.4	6.4	13.0	0.00048
**C(contrast):C(definer):C(measurer)**	1.0	4.7	4.7	9.4	0.0026
**Residual**	152.0	77.0	0.51		

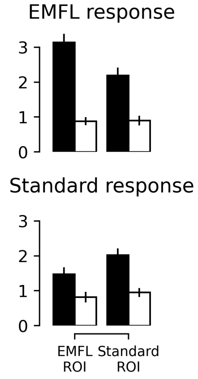

Supp. Figure 9-3: **ANOVA results and ROI-level plots for individual regions.** Bars represent beta estimates for preferred (black) and non-preferred (white, averaged across all non-preferred) conditions.

Language	df	sum_sq	mean_sq	F	PR(>F)
**C(contrast)**	1.0	29.0	29.0	89.0	5.6e-17
**C(definer)**	1.0	0.7	0.7	2.2	0.14
**C(measurer)**	1.0	0.00077	0.00077	0.0024	0.96
**C(contrast):C(definer)**	1.0	0.13	0.13	0.4	0.53
**C(contrast):C(measurer)**	1.0	1.8	1.8	5.6	0.019
**C(definer):C(measurer)**	1.0	0.17	0.17	0.52	0.47
**C(contrast):C(definer):C(measurer)**	1.0	1.6	1.6	5.1	0.026
**Residual**	152.0	49.0	0.32		

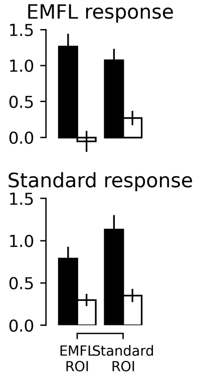

Speech	df	sum_sq	mean_sq	F	PR(>F)
**C(contrast)**	1.0	9.6	9.6	28.0	5.1e-07
**C(definer)**	1.0	4.3	4.3	13.0	0.00053
**C(measurer)**	1.0	2.8	2.8	8.1	0.005
**C(contrast):C(definer)**	1.0	0.0024	0.0024	0.0069	0.93
**C(contrast):C(measurer)**	1.0	0.54	0.54	1.6	0.21
**C(definer):C(measurer)**	1.0	1.5	1.5	4.3	0.04
**C(contrast):C(definer):C(measurer)**	1.0	0.062	0.062	0.18	0.67
**Residual**	128.0	44.0	0.34		

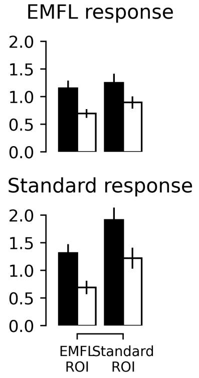

rTPJ	df	sum_sq	mean_sq	F	PR(>F)
**C(contrast)**	1.0	17.0	17.0	120.0	3.8e-21
**C(definer)**	1.0	0.0017	0.0017	0.013	0.91
**C(measurer)**	1.0	0.25	0.25	1.8	0.18
**C(contrast):C(definer)**	1.0	0.056	0.056	0.4	0.53
**C(contrast):C(measurer)**	1.0	1.1e-05	1.1e-05	8.2e-05	0.99
**C(definer):C(measurer)**	1.0	0.09	0.09	0.65	0.42
**C(contrast):C(definer):C(measurer)**	1.0	0.97	0.97	7.0	0.0089
**Residual**	152.0	21.0	0.14		

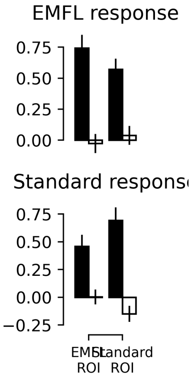

Supp. Figure 9-4: **ANOVA results and ROI-level plots for individual regions.** Bars represent beta estimates for preferred (black) and non-preferred (white, averaged across all non-preferred) conditions.

Frontal MD	df	sum_sq	mean_sq	F	PR(>F)
**C(contrast)**	1.0	17.0	17.0	70.0	3.3e-14
**C(definer)**	1.0	2.0	2.0	8.5	0.0042
**C(measurer)**	1.0	10.0	10.0	42.0	1.4e-09
**C(contrast):C(definer)**	1.0	0.057	0.057	0.24	0.63
**C(contrast):C(measurer)**	1.0	0.053	0.053	0.22	0.64
**C(definer):C(measurer)**	1.0	1.6	1.6	6.6	0.011
**C(contrast):C(definer):C(measurer)**	1.0	2.0	2.0	8.5	0.0042
**Residual**	152.0	37.0	0.24		

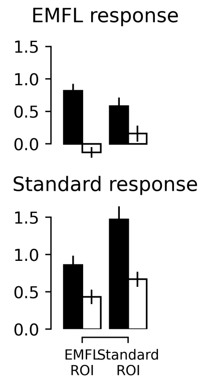

Parietal MD	df	sum_sq	mean_sq	F	PR(>F)
**C(contrast)**	1.0	38.0	38.0	73.0	1.2e-14
**C(definer)**	1.0	21.0	21.0	41.0	1.9e-09
**C(measurer)**	1.0	110.0	110.0	220.0	2.6e-31
**C(contrast):C(definer)**	1.0	0.12	0.12	0.24	0.63
**C(contrast):C(measurer)**	1.0	0.024	0.024	0.046	0.83
**C(definer):C(measurer)**	1.0	24.0	24.0	47.0	2e-10
**C(contrast):C(definer):C(measurer)**	1.0	4.5	4.5	8.7	0.0036
**Residual**	152.0	78.0	0.52		

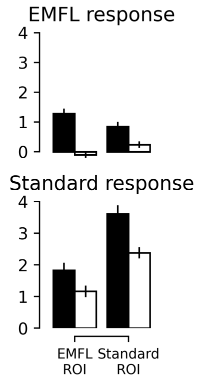

Supp. Figure 9-5: **ANOVA results and ROI-level plots for individual regions.** Bars represent beta estimates for preferred (black) and non-preferred (white, averaged across all non-preferred) conditions.

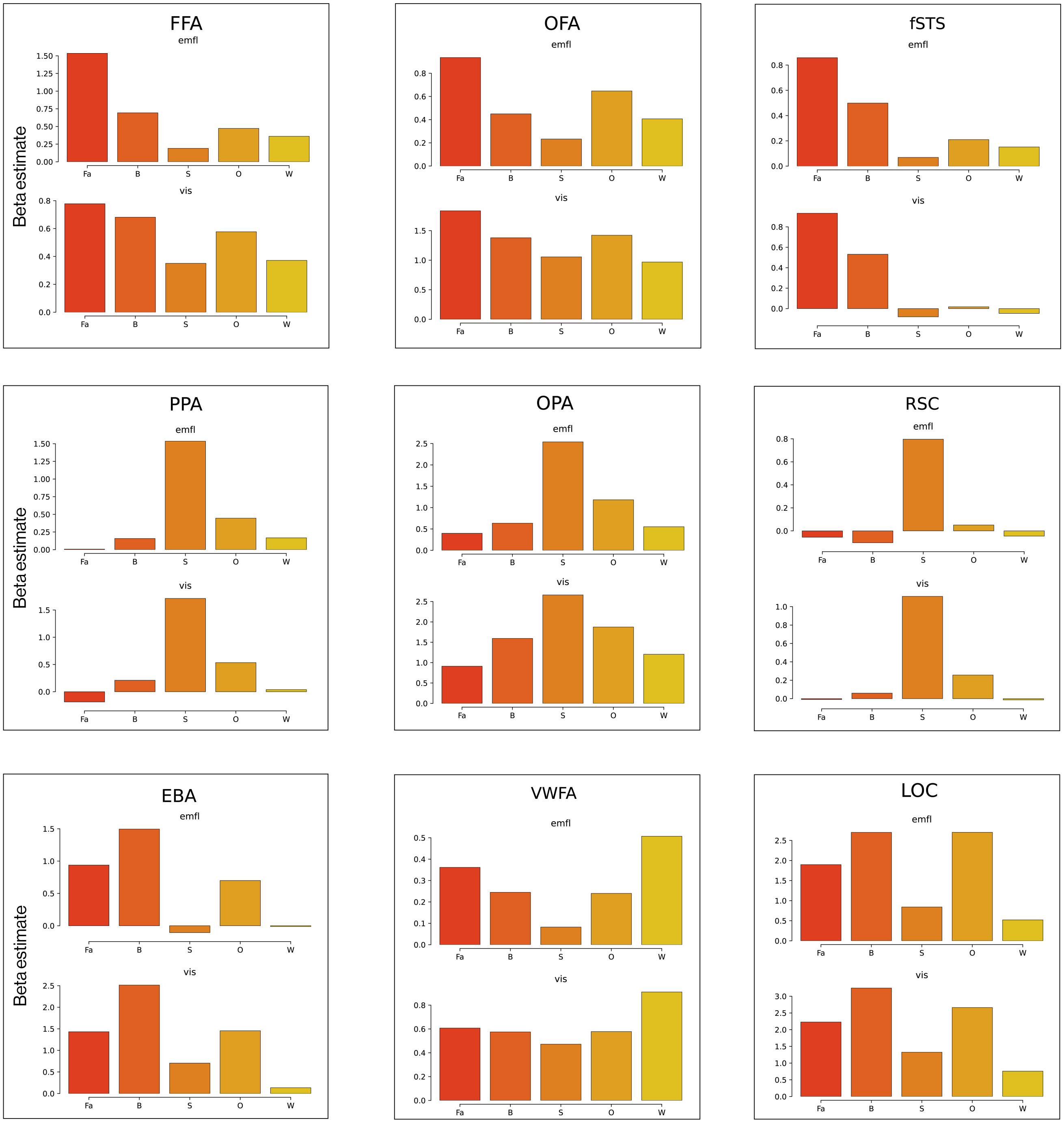

Supp. Figure 10: **Visual fROI response magnitudes to EMFL stimuli.** Five subjects were recruited for a return scan in which they viewed the EMFL stimuli without the simultaneous audio. Data from both the original EMFL with auditory task (top) and visual-only (bottom) are shown for comparison.
